# Testicular Dnmt3 expression and global DNA methylation are down-regulated by gonadotropin releasing hormones in the ricefield eel *Monopterus albus*

**DOI:** 10.1038/srep43158

**Published:** 2017-02-22

**Authors:** Yize Zhang, Xin Sun, Lihong Zhang, Weimin Zhang

**Affiliations:** 1 Institute of Aquatic Economic Animals and Guangdong Province Key Laboratory for Aquatic Economic Animals, School of Life Sciences, Sun Yat-Sen University, Guangzhou 510275, P. R. China; 2 Biology Department, School of Life Sciences, Sun Yat-Sen University, Guangzhou 510275, P. R. China

## Abstract

In vertebrates, DNA methyltransferase 3 (Dnmt3) homologues are responsible for *de novo* DNA methylation and play important roles in germ cell development. In the present study, four *dnmt3* genes, *dnmt3aa, dnmt3ab, dnmt3ba* and *dnmt3bb.1*, were identified in ricefield eels. Real-time quantitative PCR analysis showed that all four *dnmt3* mRNAs were detected broadly in tissues examined, with testicular expression at relatively high levels. In the testis, immunostaining for all four Dnmt3 forms was mainly localized to spermatocytes, which also contained highly methylated DNA. All three forms of Gonadotropin-releasing hormone (Gnrh) in the ricefield eel were shown to decrease the expression of *dnmt3* genes in the *in vitro* incubated testicular fragments through cAMP and IP_3_/Ca^2+^ pathways. Moreover, *in vivo* treatment of male fish with three forms of Gnrh decreased significantly the testicular Dnmt3 expression at both mRNA and protein levels, and the global DNA methylation levels. These results suggest that the expression of Dnmt3 and global DNA methylation in the testis of ricefield eels are potentially down-regulated by Gnrh, and reveal a novel regulatory mechanism of testicular Dnmt3 expression in vertebrates.

DNA methylation, a key epigenetic mark, participates in many physiological processes including cellular differentiation^1^, and plays crucial roles in development of vertebrates^2^. DNA methylation occurs mainly at the fifth position of cytosine (^5^mC) in the dinucleotide CpG^3^, and is catalyzed by a group of enzymes called DNA methyltransferases (Dnmts) including Dnmt1 and Dnmt3. Dnmt1 is involved in the methylation of hemimethylated DNA and thus called maintenance DNA methyltransferase, while Dnmt3 is able to place methylation marks on previously unmethylated CpGs of DNA and thus mainly responsible for the *de novo* DNA methylation during development^4^,^5^. It has been demonstrated that cellular *DNMT* expression is positively correlated with the global DNA methylation level in cell lines^6^.

Multiple *Dnmt3* genes exist in vertebrates. In mammals, *Dnmt3* subfamily was thought to be composed of three members, namely *Dnmt3a, Dnmt3b*, and *Dnmt3l*^7^. Recently, a fourth member, *Dnmt3c*, was identified^8^. Of these, Dnmt3a, Dnmt3b, and Dnmt3c have been proven to have catalytic activities *in vivo*^7^,^8^, whereas Dnmt3l is a catalytically inactive DNA methyltransferase cofactor^7^. In teleost like zebrafish, six different *dnmt3* genes are identified^9^,^10^, which are designated as *dnmt3aa, dnmt3ab, dnmt3ba, dnmt3bb.1, dnmt3bb.2*, and *dnmt3bb.3*, respectively, based on the nomenclature in ZFIN (http://zfin.org/). *Dnmt3* genes have been shown to be expressed in multiple tissues, including the testis and ovary of mouse^11^,^12^ and zebrafish^9^. During the development of male germ cells in mouse, the expression of *Dnmt3a* and *Dnmt3b* exhibits dynamic patterns^13^,^14^. *Dnmt3c* was shown to be exclusively expressed in male germ cells in mouse, and its peak expression coincided with male germline *de novo* DNA methylation^8^. These studies highlight the importance of the tightly regulated expression of *Dnmt3a, Dnmt3b*, and *Dnmt3c* during spermatogenesis. However, few studies have explored the transcriptional regulation of *Dnmt3* expression^15^. The zinc finger DNA-binding domain proteins Sp1 and Sp3 activate the transcription of human *DNMT3A* and *DNMT3B*^16^, and vascular endothelial zinc finger 1 (Vezf1) activates the transcription of mouse *Dnmt3b*^17^. The expression of *Dnmt3* has also been shown to be altered by external factors, such as the thermal stress^9^, the endocrine disrupting chemical bisphenol A^18^, and the pollutant 2,3,7,8-tetrachlorodibenzo-*p*-dioxin^19^. Interestingly, neonatal exposure to estradiol also resulted in overexpression of *Dnmt3a* and *Dnmt3b* in the prostate gland of rats^18^, suggesting that *Dnmt3* genes may be potentially subject to regulation by endocrine hormones.

The gonadotropin-releasing hormone (Gnrh), a hypothalamic decapeptide neurohormone, plays a central role in the control of vertebrate reproduction through regulation of synthesis and release of gonadotropins in the pituitary. In addition to Gnrh neurons in the hypothalamus of mammals, Gnrh has also been detected in Sertoli cells^20^ and the interstitial fluid of testis^21^, and Gnrh receptors are expressed in testicular germ cells^22^,^23^ as well. Similarly, Gnrh and Gnrh receptors have also been identified in the testis of teleosts^24^,^25^,^26^. It is suggested that local activities of Gnrh are important for testicular functions^27^. Considering the likely co-localization of Gnrh receptors and Dnmt3 in spermatogenic cells^8^,^13^,^14^,^22^,^23^, it seems intriguing to examine if Gnrh signals regulate the expression of *Dnmt3* in the testis.

The ricefield eel *Monopterus albus* is a protogynous hermaphrodite teleost that changes sex naturally from a functional female to a functional male. Previously, we have demonstrated that DNA methylation of *cyp19a1a* promoter is increased in gonads of ricefield eels during sex change towards male^28^, suggesting an important role for DNA methylation in testicular differentiation and development. In the present study, four *de novo* DNA methyltransferases, Dnmt3aa, Dnmt3ab, Dnmt3ba, and Dnmt3bb.1, were identified in the ricefield eel, and their expression was characterized at both mRNA and protein levels, particularly in the testis. It is demonstrated for the first time that Dnmt3 expression and global DNA methylation are potentially down-regulated by Gnrh signals in the testis of a vertebrate, the ricefield eel *Monopterus albus*.

## Results

### Nucleotide and deduced amino acid sequences of ricefield eel Dnmt3 homologues

Four forms of ricefield eel *dnmt3* cDNAs were obtained in the present study, which were designated as *dnmt3aa* (KX524491), *dnmt3ab* (KX524492), *dnmt3ba* (KX524493), and *dnmt3bb.1* (KX524494), respectively, based on the phylogenetic analysis (Supplementary Fig. S1) and by following the nomenclature of zebrafish *dnmt3* genes. Ricefield eel *dnmt3aa, dnmt3ab, dnmt3ba*, and *dnmt3bb.1* cDNAs encode putative proteins of 842, 990, 1484, and 825 amino acid residues, respectively.

Sequence alignment showed that all four forms of ricefield eel Dnmt3 contain the conserved putative functional domains, including one proline-tryptophan-tryptophan-proline (PWWP) motif domain, one plant homeodomain (PHD)-like Zinc finger domain, and one C-terminal catalytic domain (Supplementary Fig. S2 and S3). In contrast to the other three forms, *dnmt3ba* contains a calponin-homology (CH) domain in the N-terminal region (Supplementary Fig. S3).

### Tissue distribution patterns of *dnmt3* mRNAs in male ricefield eels

**Figure 1:**
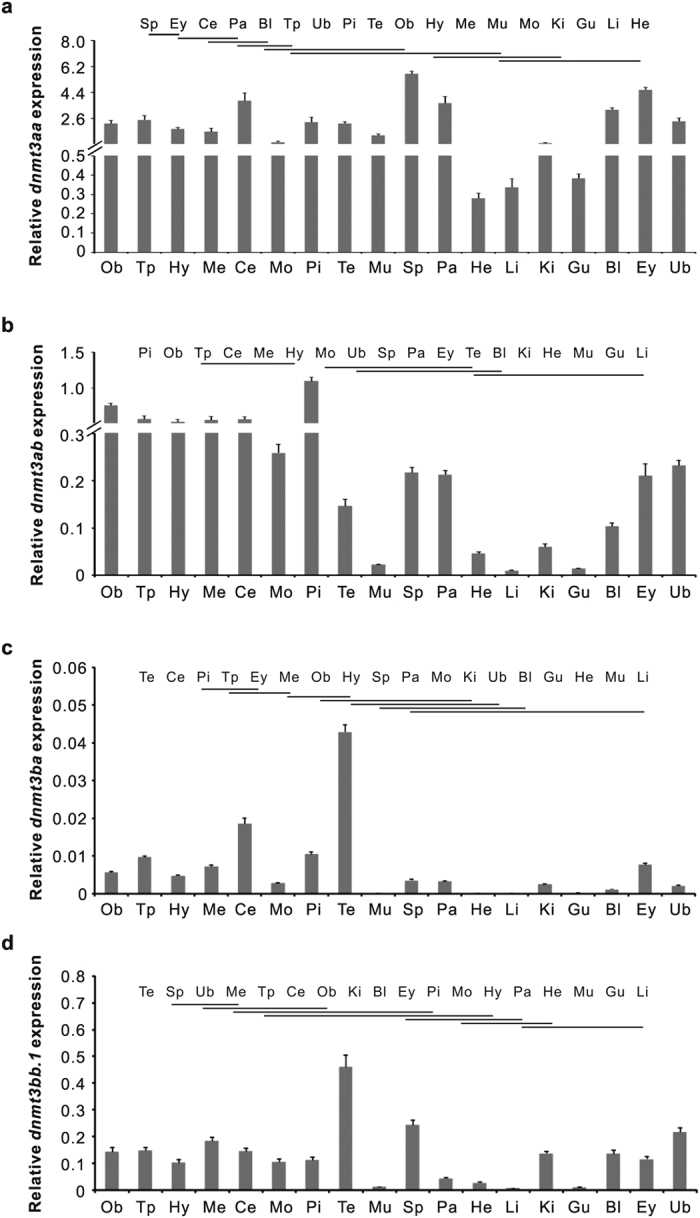
Real-time quantitative PCR analysis of *dnmt3aa* (**a**), *dnmt3ab* (**b**), *dnmt3ba* (**c**) and *dnmt3bb.1* (**d**) mRNA levels in tissues of male ricefield eels. The tissues analyzed are indicated below the corresponding bars. Bl: blood; Ce: cerebellum; Ey: eye; Gu: gut; He: heart; Hy: hypothalamus; Ki: kidney; Li: liver; Me: mesencephalon; Mo: medulla oblongata; Mu: muscle; Ob: olfactory bulb; Pa: pancreas; Pi: pituitary; Sp: spleen; Tp: telencephalon; Te: testis; Ub: urinary bladder. Each bar represents the mean of normalized expression levels ± SEM of 6 replicates. The results of statistical analysis were indicated at the top of each histogram, ranking from the high (left) to the low (right). The mRNA levels for tissues with common underscoring are not significantly different (*P* > 0.05).

The expression of *dnmt3* genes in tissues of male ricefield eels was analyzed with real-time quantitative PCR (Fig. 1). Results showed distinct tissue patterns for four *dnmt3* mRNAs in male ricefield eels, with broad distributions in tissues examined.

The expression of *dnmt3aa* was detected in all tissues examined, with relatively higher levels in the spleen, eye, cerebellum, and pancreas, and relatively lower levels in the gut, liver, and heart (Fig. 1a). The expression of *dnmt3ab* was detected at relatively higher levels in the pituitary, olfactory bulb, telencephanlon, cerebellum, mesencephalon, and hypothalamus, relatively lower levels in the medulla oblongata, urinary bladder, spleen, pancreas, eye, testis, blood, kidney, and heart, and much lower levels in the muscle, gut, and liver (Fig. 1b). The expression of *dnmt3ba* was detected at the highest level in the testis, and relatively lower levels in the spleen, pancreas, medulla oblongata, kidney, urinary bladder, and blood, but hardly detectable in the gut, heart, muscle, and liver (Fig. 1c). The expression of *dnmt3bb.1* was also detected at the highest level in the testis, and relatively lower levels in the eye, pituitary, medulla oblongata, hypothalamus, pancreas, and heart, but barely detectable in the muscle, gut, and liver (Fig. 1d).

### Cellular localization of immunoreactive Dnmt3 and global DNA methylation status in testes of ricefield eels

**Figure 2:**
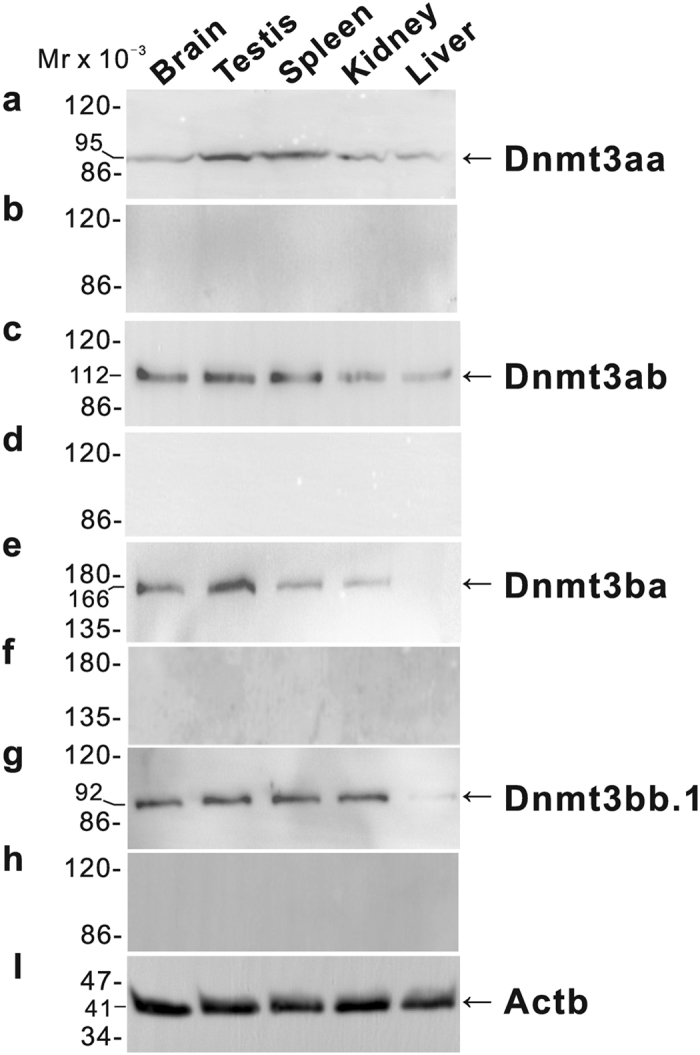
Western blot analysis of Dnmt3 proteins in tissues of male ricefield eels. The tissue homogenates from the brain, testis, spleen, kidney, and liver were separated on 8% SDS-PAGE gels, transferred to polyvinylidene difluoride membranes, and then immunoreacted with (**a**) anti-Dnmt3aa antiserum (1:1000); (**b**) anti-Dnmt3aa antiserum pre-adsorbed by excessive recombinant Dnmt3aa-N; (**c**) anti-Dnmt3ab antiserum (1:1000); (**d**) anti-Dnmt3ab antiserum pre-adsorbed by excessive recombinant Dnmt3ab-N; (**e**) antiDnmt3ba antiserum (1:1000); (**f**) anti-Dnmt3ba antiserum pre-adsorbed by excessive recombinant Dnmt3ba-N; (**g**) anti-Dnmt3bb.1 antiserum (1:1000); (**h**) anti-Dnmt3bb.1 antiserum pre-adsorbed by excessive recombinant Dnmt3bb.1-N; (**i**) mouse anti-Actb monoclonal antibody (1:500, 60008-1-Ig; ProteinTech Group, Inc. IL, USA). The secondary antibody was 1:5000 diluted horseradish peroxidase (HRP)-conjugated goat anti-mouse IgG H + L (115-035-003, Jackson ImmunoResearch Laboratories, Inc.). The blots were visualized using a BeyoECL Plus kit (Beyotime).

The specific polyclonal antisera against the ricefield eel Dnmt3aa, Dnmt3ab, Dnmt3ba, and Dnmt3bb.1 were generated, which were shown to specifically recognize the corresponding antigens, respectively (Supplementary Fig. S4). Western blot analysis indicated that Dnmt3aa and Dnmt3ab proteins were expressed at higher levels in the brain, testis, and spleen, and at lower levels in the kidney and liver (Fig. 2a and c). Dnmt3ba protein was expressed at a higher level in the testis and at lower levels in the brain, spleen, and kidney, but not detectable in the liver (Fig. 2e). Dnmt3bb.1 protein was expressed at higher levels in the brain, testis, spleen, and kidney, and at a lower level in the liver (Fig. 2g). Pre-adsorption of the antisera by excessive corresponding recombinant Dnmt3-N polypeptides abolished the immunoreactive signals in tissue homogenates (Fig. 2b,d,f and h), further confirming the specificities of the antisera generated.

**Figure 3:**
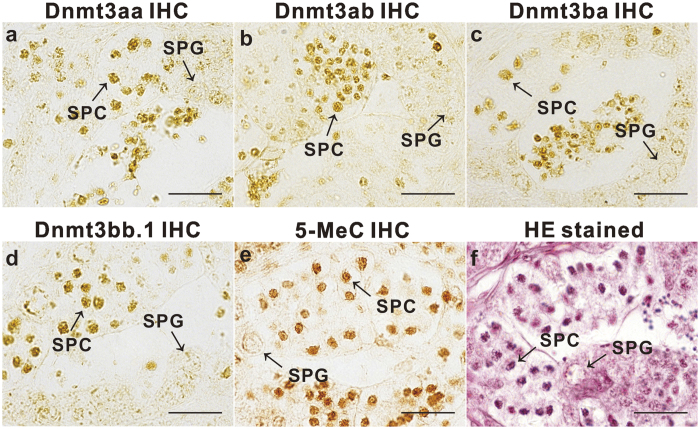
Cellular localization of immunoreactive Dnmt3 and global DNA methylation in testes of ricefield eels. Immunostaining for Dnmt3aa (**a**), Dnmt3ab (**b**), Dnmt3ba (**c**), Dnmt3bb.1 (**d**), and 5-Methylcytosine (**e**) and hematoxylin-eosin staining (**f**) were performed on sections from the same testicular tissue. The antiserum against Dnmt3aa (1:200), Dnmt3ab (1:200), Dnmt3ba (1:200), Dnmt3bb.1 (1:200) or 5-MeC (1:200) was used as the primary antiserum. The secondary antibody was 1:500 diluted horseradish peroxidase (HRP)-conjugated goat anti-mouse IgG H + L (115-035-003, Jackson ImmunoResearch Laboratories, Inc.). The immunoreactive signals were visualized by DAB chromogen. SPC, spermatocyte; SPG, spermatogonium; Scale bar = 50 μm.

The localization of the four forms of Dnmt3 in testes of ricefield eels was examined by immunohistochemistry (Fig. 3a~d). Immunostaining for all four forms of Dnmt3 was present dominantly in germ cells, particularly spermatocytes, but only weakly in spermatogonia and somatic cells of testes. The pre-adsorption of the antisera with corresponding recombinant Dnmt3-N polypeptides abolished the immunoreactive signals (Supplementary Fig. S5).

The global DNA methylation status was also examined in testes of ricefield eels by using 5-Methylcytosine (5-MeC) antibody, which could detect 5-methylcytosine but not unmethylated cytosine. Immunostaining for 5-MeC was predominantly present in germ cells, and the immunoreactive signals seem to be strong in spermatocytes but weak in spermatogonia (Fig. 3e).

### Gnrh down-regulated *dnmt3* through cAMP and IP_3_/Ca^2+^ signaling pathways in testes of ricefield eels *in vitro*

**Figure 4:**
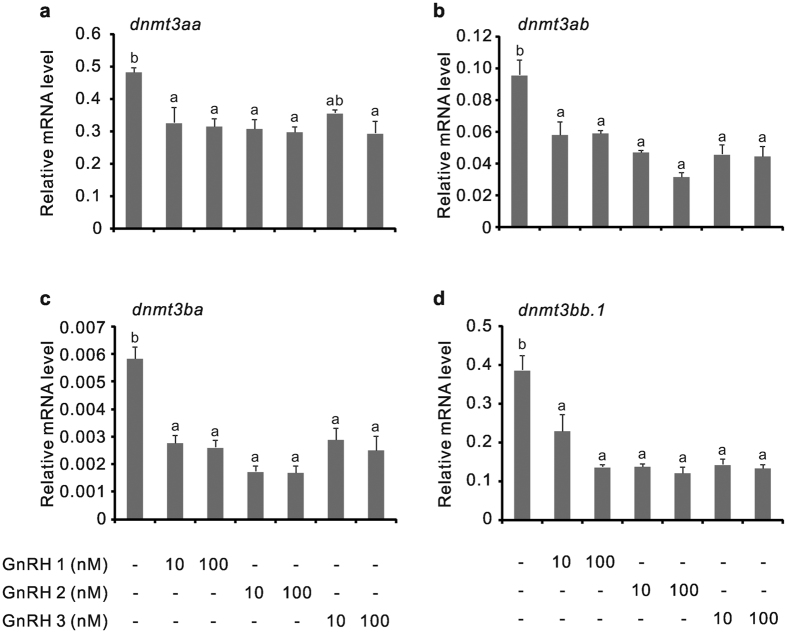
Effects of Gnrh 1, Gnrh 2 and Gnrh 3 on the expression of *dnmt3aa* (**a**), *dnmt3ab* (**b**), *dnmt3ba* (**c**) and *dnmt3bb.1* (**d**) in the *in vitro* incubated testicular fragments of male ricefield eels. The testicular fragments were pre-incubated for 18 h before treating with Gnrh 1, Gnrh 2, or Gnrh 3 (10 and 100 nM) for 8 h. After treatment, mRNA levels of *dnmt3aa, dnmt3ab, dnmt3ba*, and *dnmt3bb.1* in the testicular fragments were quantified with real-time quantitative PCR. Each bar represents the mean ± SEM of triplicates. The experiments were repeated three times, and similar results were obtained. Means marked with different letters are significantly different (*P* < 0.05).

As mRNAs for Gnrh and Gnrh receptors were detected in testes of ricefield eels (Supplementary Fig. S6), the potential involvement of Gnrh in the regulation of testicular *dnmt3* expression was examined *in vitro*. All three Gnrh forms in ricefield eels, including Gnrh 1, Gnrh 2, and Gnrh 3, could significantly down-regulate the expression of all four forms of *dnmt3* in the *in vitro* incubated testicular fragments (Fig. 4). At 100 nM, Gnrh 1 decreased the expression of *dnmt3aa, dnmt3ab, dnmt3ba*, and *dnmt3bb.1* by about 34.8%, 38.5%, 55.4%, and 64.8%, respectively; Gnrh 2 decreased the expression by about 38.5%, 67.3%, 71.3%, and 68.7%, respectively; Gnrh 3 decreased the expression by about 39.2%, 53.7%, 57.2%, and 65.6%, respectively.

**Figure 5:**
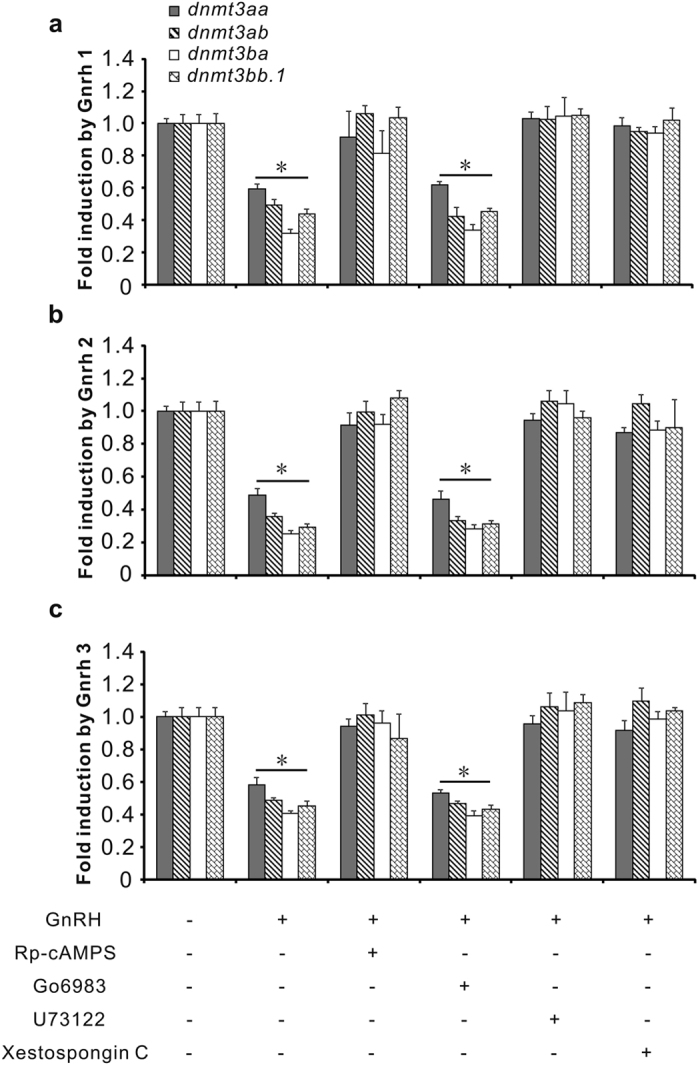
Effects of inhibitors of intracellular signaling pathways on Gnrh down-regulation of *dnmt3aa, dnmt3ab, dnmt3ba*, and *dnmt3bb.1* in the *in vitro* incubated testicular fragments of ricefield eels. The testicular fragments were pre-incubated for 18 h before treating with 100 nM of Gnrh 1 (**a**), Gnrh 2 (**b**), and Gnrh 3 (**c**) in the presence or absence of inhibitors Rp-cAMPS (50 μM), Go6983 (10 μM), U73122 (10 μM) or Xestospongin C (1 μM) respectively for 8 h. After treatment, mRNA levels of *dnmt3aa, dnmt3ab, dnmt3ba*, and *dnmt3bb.1* in the testicular fragments were quantified with real-time quantitative PCR and were presented as fold change relative to the vehicle control. Each bar represents mean ± SEM of 6 replicates. *P < 0.05 *vs* the vehicle control.

To examine the possible intracellular signaling pathways involved in the Gnrh-induced down-regulation of *dnmt3*, Rp-cAMPS (50 μM, a PKA inhibitor), Go6983 (10 μM, a PKC inhibitor), U73122 (10 μM, a PLC inhibitor), or Xestospongin C (1 μM, an IP_3_R inhibitor) was included in the *in vitro* incubated testicular fragments of ricefield eels together with Gnrh. Addition of Rp-cAMPS, U73122, or Xestospongin C abolished the inhibitory effects of Gnrh on *dnmt3* expression, however, Go6983 showed no or only minor effects (Fig. 5). Rp-cAMPS, Go6983, U73122, or Xestospongin C alone did not show any effects on *dnmt3* expression in testicular fragments of ricefield eels (Supplementary Fig. S7). Moreover, all three forms of Gnrh increased cAMP levels in the *in vitro* incubated testicular fragments (Supplementary Fig. S8). These results suggest that Gnrh down-regulates *dnmt3* expression in the testis of ricefield eels possibly through both cAMP and IP_3_/Ca^2+^ pathways but not the DAG/PKC pathway.

### Down-regulation of *dnmt3* induced by Gnrh led to reduction of global DNA methylation levels in testes of ricefield eels *in vivo*

**Figure 6:**
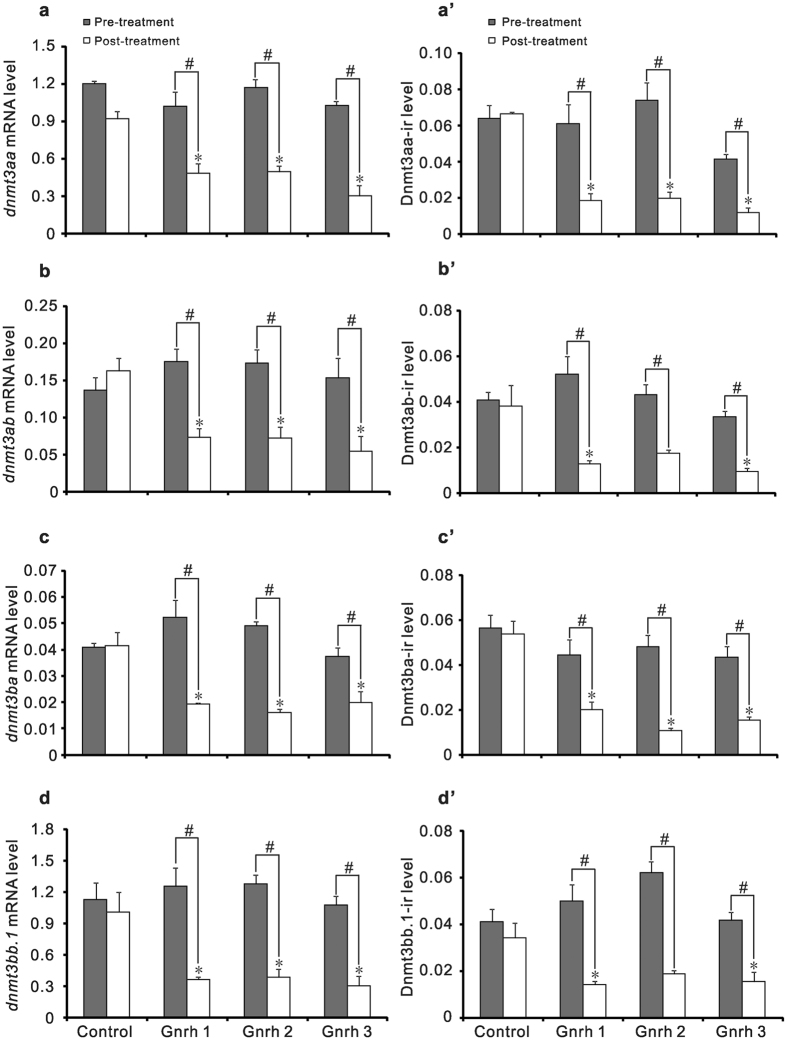
Effects of intraperitoneal injections of Gnrh 1, Gnrh 2 and Gnrh 3 on levels of *dnmt3* mRNAs (**a~d**) and Dnmt3 proteins (**a’~d’**) in testes of ricefield eels. The male ricefield eels received intraperitoneal injections of Gnrh 1, Gnrh 2, or Gnrh 3 (0.1 μg/g body weight) twice a week for four weeks. After treatments, the mRNA levels of *dnmt3aa* (**a**), *dnm3ab* (**b**), *dnmt3ba* (**c**), and *dnmt3bb.1* (**d**) in testes were quantified with real-time PCR. The immunoreactive Dnmt3aa (**a’**), Dnmt3ab (**b’**), Dnmt3ba (**c’**) and Dnmt3bb.1 (**d’**) levels in testes were analyzed by immunohistochemitry and quantified as described in Methods. Each bar represents mean ± SEM (n = 3~4). The experiments were repeated twice and similar results were obtained. *P < 0.05 *vs* the control; ^#^*P* < 0.05 for the differences between the indicated groups.

**Figure 7:**
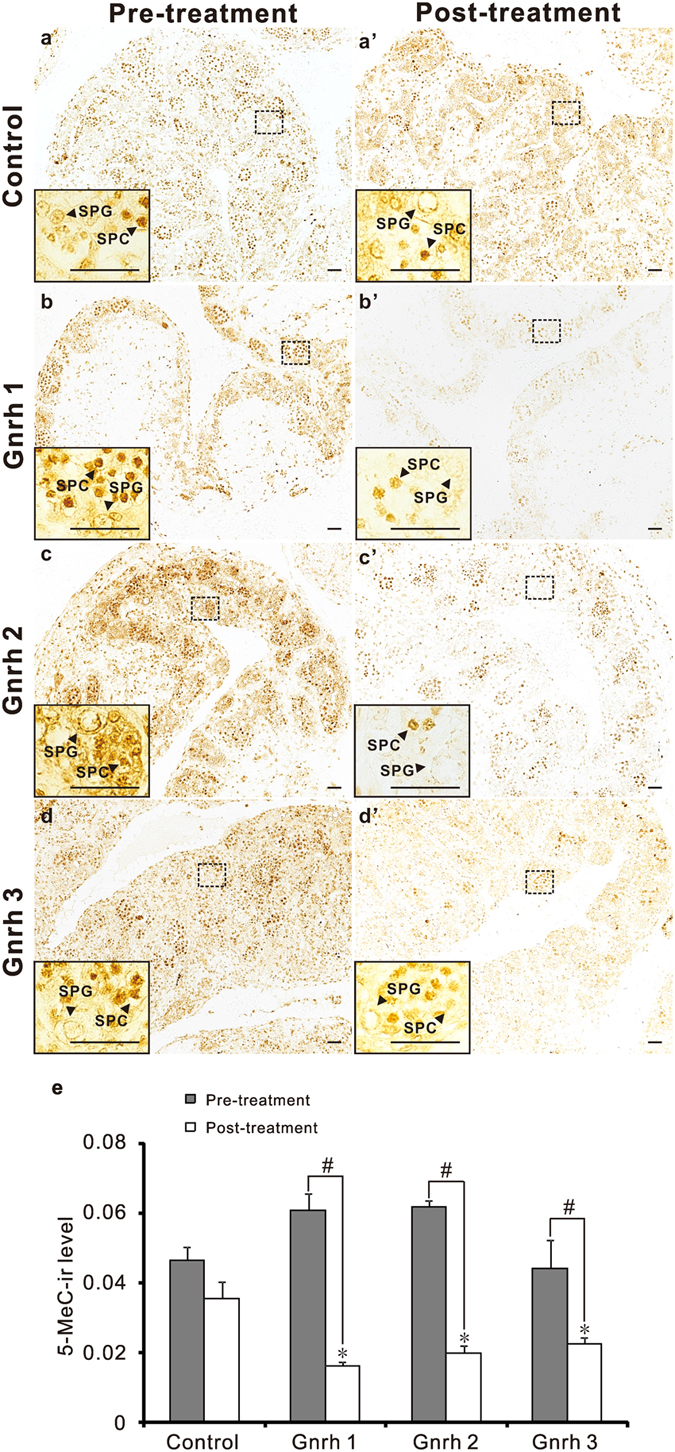
Effects of intraperitoneal injections of Gnrh 1, Gnrh 2, or Gnrh 3 on immunoreactive 5-Methylcytosine (5-MeC) levels in testes of ricefield eels. The male ricefield eels were first examined by biopsy and then received intraperitoneal injections of Gnrh 1, Gnrh 2, or Gnrh 3 (0.1 μg/g body weight) twice a week for four weeks. The 5-MeC level in the testis was analyzed with the immunohistochemistry as described in Methods, and immunoreactive signals were visualized by DAB chromogen. 5-MeC (1:200) antibody was used as the primary antibody. The secondary antibody was 1:500 diluted horseradish peroxidase (HRP)-conjugated goat anti-mouse IgG H + L (115-035-003, Jackson ImmunoResearch Laboratories, Inc.). The representative testicular sections of the same fish before (**a~d**) and after (**a’~d’**) treatments were shown, and the insets are the higher magnification of the boxed areas within each image, respectively. The immunoreactive 5-MeC levels are shown in panel e. SPC: spermatocyte; SPG: spermatogonium. Each bar in panel e represents the mean ± SEM (n = 3~4). The experiments were repeated twice and similar results were obtained. *P < 0.05 *vs* the control; ^#^*P* < 0.05 for the differences between the indicated groups. Scale bar = 25 μm.

The effects of Gnrh on *dnmt3* expression in testes of ricefield eels were also examined *in vivo* via intraperitoneal injection. The treatment with Gnrh significantly down-regulated *dnmt3* mRNA as well as Dnmt3 protein levels in testes of ricefield eels (Fig. 6; Supplementary Fig. S9). Concomitantly, the global DNA methylation levels, as reflected by immunoreactive 5-Methylcytosine levels, were also decreased significantly in testes of ricefield eels after injection of Gnrh (Fig. 7).

## Discussion

DNA methyltransferase 3 (Dnmt3) catalyzes the *de novo* DNA methylation and plays important roles in metabolism and development^5^,^29^. Multiple forms of Dnmt3 have been shown to exist in mammals^11^,^30^ and teleosts^9^,^10^,^31^,^32^. In the present study, four Dnmt3 homologues were identified in ricefield eels. Similar to those in other vertebrates, ricefield eel Dnmt3 homologues contain the conserved functional domains of DNA methyltransferases, including a catalytic domain at C-terminal, a PWWP motif domain and a plant homeodomain (PHD)-like Zn finger domain at N-terminal. Interestingly, a calponin homology (CH) domain was identified only in the N-terminus of ricefield eel Dnmt3ba, but not the other three paralogues. Similar case also exists in zebrafish^10^,^33^, but CH domain is not identified in mammalian Dnmt3 homologues^34^. CH domain belongs to a family of actin binding domains which bind to microtubules^35^. These lines of evidence suggest that teleost Dnmt3ba may possess some unique functions.

In most teleosts^9^,^32^, two *dnmt3a*-type genes (*dnmt3aa* and *dnmt3ab*) and three *dnmt3b*-type genes (*dnmt3ba, dnmt3bb.1*, and *dnmt3bb.2*) have been identified in *dnmt3* subfamily, which is probably due to the teleost-specific whole genome duplication event at the root of the crown-clade^32^. In zebrafish, *dnmt3bb.3*, a paralogue of *dnmt3bb.2*, was identified, and considered to arise from a relatively recent tandem duplication event^9^,^32^. In medaka genome, however, only three members of *dnmt3* subfamily, *dnmt3aa, dnmt3ba*, and *dnmt3bb.1*, have been identified^31^. Phylogenetic analysis categorized the four ricefield eel Dnmt3 homologues into Dnmt3aa, Dnmt3ab, Dnmt3ba, and Dnmt3bb.1, respectively. Whether ricefield eel genome contains Dnmt3bb.2 homologues as most other teleosts awaits further elucidation.

In zebrafish, RT-PCR analysis showed that two *dnmt3a* genes were ubiquitously expressed in adult tissues, while four *dnmt3b* paralogues were differentially expressed^9^. To extend this observation, our present study demonstrated distinct tissue patterns for four *dnmt3* mRNAs in male ricefield eels with real-time quantitative PCR analysis, which were further confirmed by Western blot analysis of tissue homogenates from the brain, testis, spleen, kidney, and liver. On the whole, both two *dnmt3a* genes are expressed more widely and with likely higher abundances (which was also supported by the images of agarose gel electrophoresis of semiquantitative RT-PCR products; data not shown) than *dnmt3b* genes in tissues of male ricefield eels. In most tissues of bovine, similarly, *Dnmt3a* mRNA was also detected with more higher levels than *Dnmt3b*^36^. These lines of evidence suggest that the tissue patterns of *dnmt3a* and *dnmt3b* may be conserved across vertebrates, and Dnmt3a homologues may play more important roles for *de novo* DNA methylation in more tissues of male ricefield eels and other vertebrates as well.

Significant differences in mRNA expression levels of the four *dnmt3* genes were observed among tissues in male ricefield eels, with relatively higher levels in the testis especially for *dnmt3ba* and *dnmt3bb.1*. Similarly, prominent expression of *dnmt3* was also observed in the testis of zebrafish^9^, and *dnmt3b* in the testis of mammals as well^30^. DNA methylation has important implications for gamete integrity and transmission of epigenetic information to the next generation during spermatogenesis in mammals^29^,^37^, and DNA methyltransferases are suggested to be involved in these important processes^29^,^38^. In the mouse, both Dnmt3a and Dnm3b are expressed in the male germ cells at the mRNA and protein levels^14^, and they directly interact and cooperate to establish DNA methylation patterns^38^,^39^. Consistent with these notions, both strong immunoreactive signals for Dnmt3 and prominent global DNA methylation were detected in male germ cells, particularly in spermatocytes, in the testis of ricefield eels. These results suggest that ricefield eel Dnmt3 may play conserved roles in *de novo* DNA methylation during spermatogenesis as in other vertebrates.

The expression of *Dnmt3* homologues in vertebrates has been shown to be altered by exposure to environmental toxicants, such as bisphenol A^18^, polychlorinated biphenyls^40^, and 2,3,7,8-tetrachlorodibenzo-*p*-dioxin^19^. However, the information on the regulation of *dnmt3* by physiological factors in vertebrates remains scarce. As mRNAs for three forms of Gnrh and two forms of Gnrh receptors were detected in the testis of ricefield eels (Supplementary Fig. S6), our present study proceeded to examine the possible involvement of Gnrh in the regulation of *dnmt3*. Both *in vitro* and *in vivo* treatments with Gnrh decreased *dnmt3* mRNA expression in the testis of ricefield eels. The Gnrh-induced down-regulation of *dnmt3* mRNAs was blocked by Rp-cAMPS, U73122, and Xestospongin C, but not by Go6983 in the *in vitro* incubated testicular fragments, indicating that cAMP and IP_3_/Ca^2+^ signaling pathways mediate the down-regulation of *dnmt3* by Gnrh in the testis of ricefield eels. Gnrh and/or their receptors have also been detected in the testis of many other teleosts, such as black porgy^24^ and salmon^25^. In the mature testis of rat, Gnrh mRNA was also shown to be present in Sertoli cells^41^ and the Leydig cells^23^, and Gnrhr mRNA is mainly expressed in germ cells^23^. Thus, Gnrh signals may also likely regulate *Dnmt3* expression in testes of other vertebrates, which warrants further study.

In accordance with changes at mRNA levels, immunoreactive levels of four Dnmt3 homologues were also decreased significantly in testes of ricefield eels after Gnrh treatments *in vivo*. Notably, the 5-methlcytosine levels in testes of ricefield eels were also significantly decreased after Gnrh treatments, implying a DNA hypomethylation status. Similarly, down-regulation of Dnmt1 and Dnmt3 in TK6 lymphoblastoid cell line induced by hydroquinone resulted in global DNA hypomethylation^42^, while up-regulation of *dnmt1* and *dnmt3* induced by 2,4-Dichlorophenol is associated with global DNA hypermethylation in the liver of goldfish^43^. The expression of *dnmt1* in testes of ricefield eels was not significantly decreased by Gnrh treatments in the present study (data not shown). Taken together, results of present study suggest that Gnrh induced DNA hypomethylation in testes of ricefield eels most likely through the inhibition on the expression of Dnmt3 homologues. Although posttranslational modifications may affect Dnmt3 activities^44^,^45^, the parallel decreases of immunoreactive Dnmt3, global DNA methylation, and *dnmt3* mRNA levels in the testis of ricefield eels after Gnrh treatment *in vivo* suggest that Gnrh signals regulate *Dnmt3* most likely through effects on *dnmt3* transcription and/or mRNA stability, rather than posttranslational modifications.

In the testis of vertebrates, apoptosis occurs during normal spermatogenesis^46^, and is thought to be essential for the maintenance of correct ratio of Sertoli cells and gametes^47^,^48^. Gnrh signals have been shown to induce apoptosis in the testis of immature rats^49^ and mature fish^50^,^51^,^52^, however, the underlying mechanisms remain largely unknown. In mice, intraperitoneal injection of 5-aza-2′-deoxycytidine, an inhibitor of DNA methyltransferase, led to DNA hypomethylation in spermatogonia and apoptosis in spermatogonia and spermatocytes, with prominent decreases of Dnmt3a and Dnmt3b immunoreactivities in all germ cells^53^. Decreased expression of *Dnmt3a* was also observed during apoptosis in primary cultures of rat hepatocytes^54^. Our present study demonstrated that Gnrh down-regulated Dnmt3 expression and reduced global DNA methylation levels in the testis, which may hint one possible mechanism of Gnrh induction of testicular apoptosis. Admittedly, further evidence on apoptosis in the testis of ricefield eels after Gnrh treatments, such as TUNEL staining or Caspase-3 expression, is desperately needed in this respect.

In conclusion, four Dnmt3 homologues, Dnmt3aa, Dnmt3ab, Dnmt3ba, and Dnmt3bb.1, were identified in ricefield eels, with Dnmt3aa being the most predominant form expressed in tissues examined. In the testis of ricefield eels, all four Dnmt3 homologues were shown to be localized predominantly to germ cells, particularly spermatocytes. It was shown for the first time that Gnrh treatment decreased the expression of Dnmt3 at both mRNA and protein levels, possibly through cAMP and IP_3_/Ca^2+^ signaling pathways. The results of present study may shed light on the regulation of *Dnmt3* genes in the testis of other vertebrates as well.

## Methods

### Experimental animals and tissues

Ricefield eels were obtained from a local dealer in Guangzhou, Guangdong Province, P. R. China. All procedures and investigations were reviewed and approved by the Center for Laboratory Animals of Sun Yat-Sen University, and were performed in accordance with the Guiding Principles for the Care and Use of Laboratory Animals. Fishes were sacrificed by decapitation, and tissues were dissected out, frozen immediately in liquid nitrogen, and stored at −80 °C until RNA extraction or western blot analysis. The testicular tissues for histology and immunohistochemistry were fixed in Bouin’s solution for 24 h and stored in 70% ethanol until processing. The phenotypic sex of male ricefield eels was verified by histological examination according to our previous work^55^. The testes of the male ricefield eels employed in the present study were in active spermatogenesis, with the majority of germ cells at the stage of spermatocytes.

### RNA extraction

Total RNA was isolated from frozen tissues using TRIzol (15596-026, Invitrogen MA, USA) and quantified based on the absorbance at 260 nm. The 260/280 nm ratios for all RNA samples were between 1.9 to 2.0. The integrity of RNA was checked with agarose gel electrophoresis.

### Cloning of the ricefield eel *dnmt3* cDNAs

The ricefield eel testicular cDNA was transcribed from the testis total RNA with the RevertAid H Minus First Strand cDNA Synthesis Kit (K1622, Thermo Scientific, MA, USA) according to the manufacturer’s instructions using the adapter primer AP. Two PCR products of 762 and 736 bp were generated with nested PCR, using the primer set eDnmt3-F_1_/R_1_ for the first round of amplification, and primer set eDnmt3-F_1_/R_2_ or eDnmt3-F_2_/R_1_ for the second round of amplification. The sequences of the primers are listed in Supplementary Table S1. Seven clones from each PCR product were sequenced and three different sequences were obtained, which correspond to *dnmt3aa, dnmt3ab*, and *dnmt3ba*, respectively. Then the 3′ ends of *dnmt3aa, dnmt3ab*, and *dnmt3ba* cDNAs were obtained by the RACE method using nested PCR, and the 5′ ends were extended by nested PCR using gene-specific reverse primers and degenerate forward primers targeted to conserved nucleotide sequences in 5′ utrs of *Dnmt3* homologues in other vertebrates. The cDNA sequence of *dnmt3bb.1* was initially identified from the ricefield eel pituitary transcriptome database, and then confirmed by PCR cloning from the testicular cDNA. Details are provided in Supplementary Methods.

### Real-time quantitative PCR analysis of *dnmt3* mRNA expression

Total RNA samples isolated from tissues were first treated with RNase-free DNase I (AM2222, Thermo Scientific) to remove any genomic DNA contamination. The total RNA (1 μg) was reverse transcribed with random hexamer primers using the RevertAid H Minus First Strand cDNA Synthesis Kit (K1622, Thermo Scientific) according to the manufacturer’s instruction. The integrity of all the RNA samples was verified by the successful amplification of *actb* (actin, beta; AY647143.1). Then 1 μl of cDNA template was used for the real-time quantitative PCR analysis of *dnmt3aa, dnmt3ab, dnmt3ba*, and *dnmt3bb.1* mRNA levels, respectively. The real-time quantitative PCR and quantification of mRNA expression levels were performed as previously described^56^. Details are provided in Supplementary Methods.

### Production of recombinant polypeptides and polyclonal antisera

The cDNA sequences encoding segments of ricefield eel Dnmt3aa (aa1 to 220, Dnmt3aa antigen), Dnmt3ab (aa31 to 220, Dnmt3ab antigen), Dnmt3ba (aa125 to 280, Dnmt3ba antigen), and Dnmt3bb.1 (aa1 to 209, Dnmt3bb.1 antigen) were amplified using gene-specific primers, subcloned into the expression vector pET32a (Dnmt3aa) or pET15b (Dnmt3ab, Dnmt3ba, and Dnmt3bb.1) via *Nco* I and *Bam*H I sites, and expressed in the host *E. coli* BL21 (*DE3*) as recombinant polypeptides with a TRX fusion tag (Dnmt3aa) or without fusion tags (Dnmt3ab, Dnmt3ba, and Dnmt3bb.1) by IPTG induction. The recombinant Dnmt3aa, Dnmt3ab, Dnmt3ba, and Dnmt3bb.1 antigens were purified to homogeneity, and used to immunize BALB/C mice as previously reported^57^. Details are provided in Supplementary Methods.

To examine the specificities of antisera generated, the same target polypeptides as the antigens of ricefield eel Dnmt3 homologues were also prepared with other expression vectors, including pGEX-4T-1 for Dnmt3aa (Dnmt3aa-AP) and pET32a for Dnmt3ab (Dnmt3ab-AP), Dnmt3ba (Dnmt3ba-AP), and Dnmt3bb.1 (Dnmt3bb.1-AP), respectively. These recombinant polypeptides were used as positive controls for the corresponding anti-Dnmt3 antiserum in western blot analysis. Moreover, the N-terminal regions of Dnmt3aa (aa1 to 525, Dnmt3aa-N), Dnmt3ab (aa1 to 528, Dnmt3ab-N), Dnmt3ba (aa1 to 486, Dnmt3ba-N), and Dnmt3bb.1 (aa1 to 476, Dnmt3bb.1-N), which encompass the antigen regions, were also prepared with the expression vector pGEX-4T-1. Details are provided in Supplementary Methods. The purified recombinant Dnmt3aa-N, Dnmt3ab-N, Dnmt3ba-N, and Dnmt3bb.1-N polypeptides was employed in western blot and immunohistochemical analysis to further validate the specificities of anti-Dnmt3 antisera generated.

### Western blot analysis

The recombinant proteins or tissue homogenates (300 μg) were separated on a 8% SDS-PAGE gel and transferred to a methanol-activated polyvinylidene difluoride membrane (ISEQ00010, Merck Millipore, MA, USA) by electroblotting. The membrane was then blocked with 5% nonfat milk powder in 10 mM PBS buffer (137 mM NaCl, 2.7 mM KCl, 10 mM Na_2_HPO_4_, 2 nM KH_2_PO_4_) at 4 °C overnight. The blocked membrane was then incubated sequentially with the primary antiserum and second antibody, and exposed to a chemiluminescence substrate (BeyoECL Plus kit, P0018, Beyotime, Shanghai, China) according to the manufacturer’s instructions. Details are provided in Supplementary Methods.

### Immunohistochemistry

The testicular sections (5 μm) were deparaffinized, hydrated, and incubated with 3% hydrogen peroxide solution to quench the endogenous peroxidase activity, followed by antigen retrieval in 10 mM citrate buffer (pH = 6.0) at 95 °C for 15 min and blocking in 0.01 M PBS containing 10% normal goat serum for 30 min at room temperature. Then the sections were incubated with the primary mouse anti-Dnmt3aa, anti-Dnmt3ab, anti-Dnmt3ba or anti-Dnmt3bb.1 antiserum (1:200) at 4 °C overnight. After rinsing with PBS for 5 min three times, the sections were exposed to the secondary antibody (HRP-conjugated goat anti-mouse IgG, 1:500 dilution; 115-035-003, Jackson ImmunoResearch Laboratories, Inc., PA, USA) solution. After rinsing with PBS, the sections were developed with 3,3′-diaminobenzidine (DAB), mounted, examined with a Nikon Eclipse Ni-E microscope (Nikon, Japan), and digitally photographed. To confirm the specificity of the immunostaining, control sections were incubated with the primary antiserum (in its working solution) pre-adsorbed with an excess of corresponding recombinant Dnmt3-N polypeptides. Additional negative controls included replacement of the primary antiserum with PBS or pre-immune serum and the omission of secondary antibody.

The assessment of global DNA methylation in the testis of ricefield eel was performed with immunohistochemistry using the anti-5-methylcytosine antibody (MABE146, Merck Millipore) according to a previous report^58^, and details are provided in Supplementary Methods.

The immunoreactive levels of 5-Methylcytosine and Dnmt3 in the testes were analyzed with the Image Pro Plus software (Media Cybernetics, Inc., MD, USA) in a way similar to a previous report^59^. All the sections and photoimages for analysis were processed under the same conditions. The measurement for each fish was based on three sections at an interval of about 100 μm, and at least three fish samples were analyzed for each group. The data are presented as means + SEM (n = 3~4). Details are provided in Supplementary Methods.

### *In vitro* treatment of testicular fragments with Gnrh

The testicular tissues of male ricefield eels were dissected out and chopped into pieces of approximately 1 mm^3^ with a scalpel. Approximately 25 mg of testicular minces were placed in each well of a 24-well tissue culture plate (142475, Nunc, Denmark) with 500 μl of L15 medium (11415064, Gibco, MA, USA) containing 0.1 U/ml penicillin and 0.1 μg/ml streptomycin (15140163, Gibco), and then incubated at 28 °C in a humidified incubator (SPX-250BSH-II, CIMO, Shanghai, China). After pre-incubation for 18 h, the medium was replaced and the testicular fragments were treated with 10 and 100 nM of ricefiel eel Gnrh 1 (pjGnrh; AAW51121), Gnrh 2 (cGnrh-II; AAW51119), or Gnrh3 (sGnrh; AAW51120), either in the absence or presence of the PKA inhibitor Rp-cAMPS (50 μM; sc-24010, Santa Cruz, TX, USA), the PKC inhibitor Go6983 (10 μM; S2911, Selleckchem, TX, USA), the PLC inhibitor U73122 (10 μM; S8011, Selleckchem,), or the IP_3_R inhibitor Xestospongin C (1 μM; 1280, TOCRIS, Bristol, UK) for 8 h. Six replicates were performed for each treatment except for Gnrh dose response, where the experiments were repeated three times with three replicates for each treatment. After completion of incubation, tissues were collected and expression of *dnmt3aa, dnmt3ab, dnmt3ba*, and *dnmt3bb.1* was analyzed with real-time quantitative PCR as described above. The experiments were repeated at least three times, and similar results were obtained.

### *In vivo* treatments of male fish with Gnrh

Presumably male ricefield eels (body length 35~45 cm, body weight 50~80 g) were purchased from a local dealer in Guangzhou, Guangdong, P. R. China, kept in 50-litre plastic tanks in laboratory under a natural photoperiod (23.11 °N) and temperature (25.1 ± 1.0 °C) from October to November, 2015, and fed live *Tenebrio molitor*. The tank water was replaced on alternate days.

After acclimatization for one week, ricefield eels were biopsied to identify their sex in a way similar to a previous report^60^, and only males were included in this study. One week after biopsy, male ricefield eels received intraperitoneal injections of Gnrh1, Gnrh2, and Gnrh3 at 0.1 μg/g body weight or physiological saline (control) twice a week for four weeks. The dose of Gnrh was determined according to the previous report^60^ and the results of the pilot experiments (data not shown). Twenty four hours after the last injection, the testicular tissues of ricefield eels were dissected out, and the expression of Dnmt3 homologues as well as the DNA methylation status were analyzed as above.

### Statistical analysis

Data were analyzed by one-way ANOVA followed by the Tukey multiple comparison test using the SPSS 17.0 software (SPSS, Inc., NY, USA). Significance was set at *P* < 0.05.

## Additional Information

**How to cite this article:** Zhang, Y. *et al*. Testicular Dnmt3 expression and global DNA methylation are down-regulated by gonadotropin releasing hormones in the ricefield eel *Monopterus albus. Sci. Rep.* **7**, 43158; doi: 10.1038/srep43158 (2017).

**Publisher's note:** Springer Nature remains neutral with regard to jurisdictional claims in published maps and institutional affiliations.

## Supplementary Material

Supplementary Information
