# Supplementary material for: Testicular Dnmt3 expression and global DNA methylation are down-regulated by gonadotropin releasing hormones in the ricefield eel Monopterus albus
Source: Sci Rep. 2017 Feb 22;7:43158. doi: 10.1038/srep43158 (PMC5320511; doi:10.1038/srep43158)
Supplement: Supplementary Information [file srep43158-s1.pdf]

Supplementary Information

Testicular Dnmt3 expression and global DNA methylation are down-regulated by gonadotropin releasing hormones in the ricefield eel *Monopterus albus*

Yize Zhang[1], Xin Sun[1], Lihong Zhang[2], Weimin Zhang[1,2]

[1]Institute of Aquatic Economic Animals and Guangdong Province Key Laboratory for Aquatic Economic Animals, School of Life Sciences;

[2]Biology Department, School of Life Sciences, Sun Yat-Sen University, Guangzhou 510275, P. R. China

Correspondence:

Weimin Zhang, Institute of Aquatic Economic Animals, School of Life Sciences, Sun Yat-Sen University, Guangzhou 510275, P. R. China; e-mail: lsszwm@mail.sysu.edu.cn; Tel: 86-20-84113327; or Lihong Zhang, Biology Department, School of Life Sciences, Sun Yat-Sen University, Guangzhou 510275, P. R. China; e-mail: zhlih@mail.sysu.edu.cn; Tel: 86-20-8411- 0828; Fax: 86-20-84113327.

Supplementary Methods

Cloning of the ricefield eel *dnmt3* cDNAs.

The ricefield eel testicular cDNA was transcribed from the testis total RNA with the RevertAid H Minus First Strand cDNA Synthesis Kit (K1622, Thermo Scientific, MA, USA) according to the manufacturer's instructions using the adapter primer AP, and then 1 μl of the testicular cDNA was amplified by nested PCR, using the primer set eDnmt3-F_1/R_1 for the first round of amplification, and primer set eDnmt3-F_1/R_2 or eDnmt3-F_2/R_1 for the second round of amplification. PCR was performed in a 25-μl final volume containing 2.5 μl 10x *Taq* Buffer, 2.5 mM $MgCl_2$, 0.2 mM dNTP, 0.4 μM of each primer, and 1.25 U Fermentas *Taq* DNA Polymerase (EP0402, Fermentas, MA, USA). After an initial 3 min denaturing step at 94 °C, 35 cycles of amplification were performed with 0.5 min at 94 °C, 0.5 min at 48 (first) or 50 (second) °C, and 1.5 min at 72 °C, and then followed by a final extension for 10 min at 72 °C using the TGRADIENT thermal cycler (Biometra GmbH, Goettingen, Germany). PCR products of 762 and 736 bp were generated with primer sets eDnmt3-F_1/R_2 and eDnmt3-F_2/R_1, respectively. The primers were targeted to the nucleotide sequences in highly conserved regions of previously identified vertebrate *Dnmt3* homologues. Seven clones from each PCR product were sequenced and three different sequences were obtained which correspond to *dnmt3aa* (762 bp), *dnmt3ab* (736 bp), and *dnmt3ba* (736 bp), respectively. Then the 3' ends of *dnmt3aa*, *dnmt3ab*, and *dnmt3ba* cDNAs were obtained by the RACE method using nested PCR. The primers were eDnmt3aa-F_1 and BRL-A_2 for the first round and eDnmt3aa-F_2 and BRL-A_2 for the second round of amplification of *dnmt3aa*, eDnmt3ab-F_1 and BRL-A_2 for the first round and eDnmt3ab-F_2 and BRL-A_2 for the second round of amplification of *dnmt3ab*,

and eDnmt3ba-F_1 and BRL-A_2 for the first round and eDnmt3ba-F_2 and BRL-A_2 for the second round of amplification of *dnmt3ba*. These primers were listed in Supplementary Table S1. The cycling conditions were the same as the above except that annealing temperature was 58 °C. The target PCR products were processed and analyzed as above.

The 5' ends of *dnmt3aa*, *dnmt3ab*, and *dnmt3ba* cDNAs were extended by nested PCR using gene-specific reverse primers and degenerate forward primers targeted to conserved nucleotide sequences in 5' utrs of *Dnmt3* homologues in other vertebrates. The 5' ends of *dnmt3aa* and *dnmt3ab* were extended for two times with nested PCR. In the first extension, the primers were eDnmt3aa-F_3 and eDnmt3aa-R_1 for the first PCR amplification and eDnmt3aa-F_3 and eDnmt3aa-R_2 for the second PCR amplification of *dnmt3aa*, and eDnmt3ab-F_3 and eDnmt3ab-R_1 for the first PCR amplification and eDnmt3ab-F_3 and eDnmt3ab-R_2 for the second PCR amplification of *dnmt3ab*. In the second extension, the primers were eDnmt3aa-F_4 and eDnmt3aa-R_3 for the first PCR amplification and eDnmt3aa-F_4 and eDnmt3aa-R_4 for the second PCR amplification of *dnmt3aa*, and eDnmt3ab-F_4 and eDnmt3ab-R_3 for the first PCR amplification and eDnmt3ab-F_4 and eDnmt3ab-R_4 for the second amplification of *dnmt3ab*. The 5' end of *dnmt3ba* was extended for three times with nested PCR. In the first extension, the primers were eDnmt3ba-F_3 and eDnmt3ba-R_1 for the first PCR amplification and eDnmt3ba-F_3 and eDnmt3ba-R_2 for the second PCR amplification. In the second extension, the primers were eDnmt3ba-F_4 and eDnmt3ba-R_3 for the first PCR amplification and eDnmt3ba-F_4 and eDnmt3ba-R_4 for the second PCR amplification. In the third extension, the primers were eDnmt3ba-F_5 and eDnmt3ba-R_5 for the first PCR amplification and eDnmt3ba-F_5 and eDnmt3ba-R_6 for second

PCR amplification. The cycling conditions were the same as obtaining the initial cDNA fragments, and the target PCR products were processed and analyzed as above.

The cDNA sequence of *dnmt3bb.1* was initially identified from the ricefield eel pituitary transcriptome database, and then confirmed by PCR cloning from the testicular cDNA. The primers were eDnmt3bb.1-F and eDnmt3bb.1-R, and the cycling conditions were the same as obtaining the 3' ends. The target PCR products were processed and analyzed as above. The sequences of all the primers are listed in Supplementary Table S1.

Real-time quantitative PCR analysis of dnmt3 mRNA expression.

Total RNA samples isolated from tissues were first treated with RNase-free DNase I (AM2222, Thermo Scientific) to remove any genomic DNA contamination. The total RNA (1 μg) was reverse transcribed with random hexamer primers using the RevertAid H Minus First Strand cDNA Synthesis Kit (K1622, Thermo Scientific) according to the manufacturer's instruction. The integrity of all the RNA samples was verified by the successful amplification of *actb* (actin, beta; AY647143.1). Real-time quantitative PCR analysis was established as previously described[1] to determine the mRNA expression levels of ricefield eel *dnmt3*. Three reference genes, namely *actb*, *gapdh*, *hprt1*, were employed in the present study to minimize the potential inaccuracy when using a single reference gene. The primers were qrt-ednmt3aa-F and qrt-ednmt3aa-R for *dnmt3aa*, qrt-ednmt3ab-F and qrt-ednmt3ab-R for *dnmt3ab*, qrt-ednmt3ba-F and qrt-ednmt3ba-R for *dnmt3ba*, qrt-ednmt3bb.1-F and qrt-ednmt3bb.1-R for *dnmt3bb.1*, b-actin-qF_2 and b-actin-qR_2 for *actb*, gapdh-qF and gapdh-qR for glyceraldehyde-3-phosphate dehydrogenase gene (*gapdh*; FJ873738.1),

hprt1-qF and hprt1-qR for hypoxanthine phosphoribosyltransferase 1 gene (*hprt1*; DQ218476.1). The sense and antisense primers for real-time quantitative PCR analysis were targeted to different exons respectively in order to discern possible amplification of any potential contaminating genomic DNA. The nucleotide sequences of primers are listed in Supplementary Table S2.

The real-time quantitative PCR was performed on the iCycler iQ5 (Bio-Rad) in a volume of 20 μl containing 0.2 μM of each primer, 10 μl of 2X SYBR Green Master Mix (QPK-201, TOYOBO, Osaka, Japan), and 1 μl of cDNA template which was reverse transcribed as above. The PCR cycling conditions were: 95 °C for 3 min; 40 cycles of 95 °C for 15 s, 58 °C for 15 s, 72 °C for 15 s; 82 °C for 15 s for signal collection in each cycle. Data were produced and analyzed by iQ5 software. The specificity of PCR amplification was confirmed by melt-curve analysis, agarose gel electrophoresis, and sequencing of PCR products. All samples were run in duplicates and minus reverse transcriptase and no template controls were included in all plates.

The quantification of the mRNA expression level was performed using a standard curve with tenfold serial of dilution of plasmid containing corresponding DNA fragments from 10^1 to 10^8 copies. The correlation coefficients and PCR efficiencies were not less than 0.98 and 95%, respectively. The copy numbers of *dnmt3* and reference genes were calculated by iQ5 software (Bio-rad) based on the corresponding standard curves. To minimize variation due to the differences in RNA loading, each sample was normalized to the geometric mean of the expression levels of the three reference genes, and the mRNA expression levels of *dnmt3* were presented as the copy number ratios to the geometric means of reference genes.

Production of recombinant polypeptides and polyclonal antisera.

The cDNA sequences encoding segments of ricefield eel Dnmt3aa (aa1 to 220, Dnmt3aa antigen), Dnmt3ab (aa31 to 220, Dnmt3ab antigen), Dnmt3ba (aa125 to 280, Dnmt3ba antigen), and Dnmt3bb.1 (aa1 to 209, Dnmt3bb.1 antigen) were amplified using gene-specific primer sets eDnmt3aa-pET-F/eDnmt3aa-pET-R, eDnmt3ab-pET-F/eDnmt3ab-pET-R, eDnmt3ba-pET-F /eDnmt3ba-pET-R, and eDnmt3bb.1-pET-F/eDnmt3bb.1-pET-R, respectively. The sequences of the primers were listed in Supplementary Table S2. The cDNA fragments were subcloned into the expression vector pET32a (Dnmt3aa) or pET15b (Dnmt3ab, Dnmt3ba and Dnmt3bb.1) via *Nco* I and *Bam*H I sites, and expressed in the host *E. coli* BL21 (*DE3*) as recombinant polypeptides with a TRX fusion tag (Dnmt3aa) or without fusion tags (Dnmt3ab, Dnmt3ba, and Dnmt3bb.1) by IPTG induction for 3 h. SDS-PAGE analysis showed that target proteins for recombinant Dnmt3ab, Dnmt3ba, and Dnmt3bb.1, and Dnmt3aa were produced only by the corresponding expression constructs after IPTG induction but not by the empty expression vectors. The recombinant Dnmt3ab, Dnmt3ba, and Dnmt3bb.1 antigens were shown to be present in inclusion bodies. These inclusion bodies were first washed with inclusion wash buffer (2 M Urea, 50 mM Tris, 50 mM NaCl, 1 mM EDTA, 1% Triton-X 100, pH8.0) and then separated on 12% SDS-PAGE gels. The target bands were cut out from the gel after staining with 0.5 M KCl, extracted in ultrapure water at 4 ℃ overnight, and purified to homogeneity based on SDS-PAGE analysis. While the recombinant Dnmt3aa antigen was shown to be present in soluble form. The soluble recombinant Dnmt3aa was first substantially purified by using the His Bind Purification Kit

(70239-3, Merck Millipore, MA, USA) and then further gel purified as described above. The purified Dnmt3 antigens were used to immunize BALB/C mice as previously reported[2]. Briefly, the purified Dnmt3 antigen was mixed with an equal volume of Freund adjuvant (F5881, Sigma, Munich, Germany) and injected into BALB/C mice subcutaneously at 7-day intervals. The mouse antisera were collected on the seventh day after the fourth immunization.

To examine the specificities of antisera generated, the same target polypeptides as the antigens of ricefield eel Dnmt3 homologues were also prepared with other expression vectors, including pGEX-4T-1 for Dnmt3aa (Dnmt3aa-AP) and pET32a for Dnmt3ab (Dnmt3ab-AP), Dnmt3ba (Dnmt3ba-AP), and Dnmt3bb.1 (Dnmt3bb.1-AP), respectively. The recombinant polypeptides were overexpressed in inclusion bodies in the host *E. coli* BL21 (*DE3*), purified as described above, and used as positive controls for the corresponding antiserum in Western blot analysis. Furthermore, the cDNA sequences encoding the N-terminal regions of Dnmt3aa (aa1 to 525, Dnmt3aa-N), Dnmt3ab (aa1 to 528, Dnmt3ab-N), Dnmt3ba (aa1 to 486, Dnmt3ba-N), and Dnmt3bb.1 (aa1 to 476, Dnmt3bb.1-N) were also amplified with primer sets Exp-ednmt3aa-F/Exp-ednmt3aa-R, Exp-ednmt3ab-F/ Exp-ednmt3ab-R, Exp-ednmt3ba-F/Exp-ednmt3ba-R and Exp-ednmt3bb.1-F/ Exp-ednmt3bb.1-R respectively, and subcloned into the expression vector pGEX-4T-1. The sequences of primers are listed in Supplementary Table S2. After IPTG induction, recombinant Dnmt3aa-N, Dnmt3ab-N, Dnmt3ba-N, and Dnmt3bb.1-N were overexpressed in inclusion bodies in the host *E. coli* BL21 (*DE3*) and processed as above. The purified recombinant Dnmt3aa-N, Dnmt3ab-N, Dnmt3ba-N, and Dnmt3bb.1-N polypeptides was employed in western blot and

immunohistochemical analysis to further validate the specificities of anti-Dnmt3 antisera generated.

Western blot analysis.

The recombinant proteins or tissue homogenates (300 μg) were separated on a 8% SDS-PAGE gel and transferred to a methanol-activated polyvinylidene difluoride membrane (ISEQ00010, Merck Millipore) by electroblotting. The membrane was then blocked with 5% nonfat milk powder in 10 mM PBS buffer (137 mM NaCl, 2.7 mM KCl, 10 mM Na_2HPO_4, 2 nM KH_2PO_4) at 4 ℃ overnight. The anti-Dnmt3aa, Dnmt3ab, Dnmt3ba or Dnmt3bb.1 antiserum was pre-adsorbed for 4 h at 4 ℃ with extracts of *E.coli* BL21 (*DE3*) bacteria that were transformed with the empty vector pET32a or pET15b induced by IPTG. As negative controls for specificities, the anti-Dnmt3 antisera were further pre-adsorbed with corresponding recombinant Dnmt3-N polypeptides. The blocked membrane was then incubated with the pre-adsorbed anti-Dnmt3aa, Dnmt3ab, Dnmt3ba or Dnmt3bb.1 (1:1000), or beta Actin Mouse Monoclonal antibody (1:500, 60008-1-Ig; ProteinTech Group, Inc., IL, USA) in blocking solution (5% nonfat milk powder in 10 mM PBS) at room temperature for 4 h, washed with PBS for 5 min three times, and incubated with horseradish peroxidase (HRP)-conjugated goat anti-mouse immunoglobulin G (IgG) (1:5000; 115-035-003, Jackson ImmunoResearch Laboratories, Inc., PA, USA) for 1 h at room temperature. After three 10 min final washes with PBS, the membranes were exposed to a chemiluminescence substrate (BeyoECL Plus kit, P0018, Beyotime, Shanghai, China) according to the manufacturer's instructions.

Immunohistochemistry.

The testicular sections (5 μm) were deparaffinized, hydrated, and incubated with 3% hydrogen peroxide solution to quench the endogenous peroxidase activity, followed by antigen retrieval in 10 mM citrate buffer (pH=6.0) at 95 ℃ for 15 min and blocking in 0.01 M PBS containing 10% normal goat serum for 30 min at room temperature. Then the sections were incubated with the primary mouse anti-Dnmt3aa, anti-Dnmt3ab, anti-Dnmt3ba or anti-Dnmt3bb.1 antiserum (1:200) at 4 ℃ overnight. After rinsing with PBS for 5 min three times, the sections were exposed to the secondary antibody (HRP-conjugated goat anti-mouse IgG, 1:500 dilution; 115-035-003, Jackson ImmunoResearch Laboratories, Inc.) solution. After rinsing with PBS, the sections were developed with 3,3'-diaminobenzidine (DAB), mounted, examined with a Nikon Eclipse Ni-E microscope (Nikon, Japan), and digitally photographed. To confirm the specificity of the immunostaining, control sections were incubated with the primary antiserum (in its working solution) pre-adsorbed with an excess of corresponding recombinant Dnmt3-N polypeptides. Additional negative controls included replacement of the primary antiserum with PBS or pre-immune serum and the omission of secondary antibody.

The assessment of DNA methylation status in the testis of ricefield eel was performed according to a previous report[3]. Briefly, the sections were deparaffinized and hydrated as described above, then incubated in 2 N HCl for 2 h at 37 ℃ to hydrolyze DNA and increase the antigen accessibility. After being washed in 0.01 M PBS, the sections were covered with the anti-5-methylcytosine antibody (MABE146, Merck Millipore) at 10 μg/ml and incubated

overnight at 4 ℃. After rinsing with PBS for 5 min three times, the sections were incubated with the secondary antibody as described above, and followed by washing in PBS again and in 3% hydrogen peroxide for 15 min to block the endogenous peroxidase activity. The detection and acquisition of the signals were performed as described above.

The immunoreactive levels of 5-Methylcytosine and Dnmt3 in the testes were analyzed with the Image Pro Plus software (Media Cybernetics, Inc., MD, USA) in a way similar to a previous report[4]. All the sections and photoimages for analysis were processed under the same conditions. Briefly, the whole gonadal lamellae (20× magnification) was defined as the region of interest (ROI). For each ROI, the immunoreactive 5-Methylcytosine or Dnmt3 expression was shown as DAB staining in gonadal lamellae. Within the gonadal lamellae, the sum integrated optical density (IOD) value of DAB staining and area of total gonadal lamellae were calculated using the "Count and Measure" tools of Image Pro Plus software. The mean density for 5-Methylcytosine or Dnmt3 in each ROI, which corresponds to the immunoreactive level, was defined as the sum IOD divided by the sum area of the gonadal lamellae. The measurement for each fish was based on three sections at an interval of about 100 μm, and at least three fish samples were analyzed for each group. The data are presented as means ± SEM (n=3~4).

Assay of Gnrh-induced production of cAMP in testicular fragments of ricefield eels.

The testicular tissues of male ricefield eels were dissected out and chopped into pieces of approximately 1 mm^3 with a scalpel. Approximately 40 mg of testicular minces were placed in each well of a 24-well tissue culture plate (142475, Nunc, Denmark) with 500

μl of L15 medium (11415064, Gibco, MA, USA) containing 0.1 U/ml penicillin and 0.1 μg/ml streptomycin (15140163, Gibco), and then incubated at 28 ℃ in a humidified incubator (SPX-250BSH-II, CIMO, Shanghai, China). After pre-incubation for 18 h, the medium was replaced and the testicular fragments were treated with 10 and 100 nM of ricefiel eel Gnrh 1 (pjGnrh; AAW51121), Gnrh 2 (cGnrh-II; AAW51119), or Gnrh3 (sGnrh; AAW51120) for 8 h. Each treatment was repeated in six wells. After treatment, testicular fragments were collected, frozen immediately in liquid nitrogen, and ground to a fine powder under liquid nitrogen in a stainless steel mortar. After evaporation of the liquid nitrogen, the frozen tissues were weighed and homogenized in 10 volumes of 0.1M HCl. After Centrifugation at 1000 x g for 5 min at room temperature, the supernatant testicular homogenate was collected. cAMP concentrations in testicular homogenates were assayed with a Monoclonal Anti-cAMP Antibody Based Direct cAMP ELISA Kit (New Non-acetylated Version; NewEast Biosciences, Inc., PA, USA) by following manufacturer's instructions. Results are expressed as fold induction relative to the vehicle control. The data are presented as means $\pm$ SEM (n=6).

References

1 He, Z. *et al.* Growth differentiation factor 9 (Gdf9) was localized in the female as well as male germ cells in a protogynous hermaphroditic teleost fish, ricefield eel *Monopterus albus*. *Gen Comp Endocrinol* **178**, 355-362, (2012).

2 Wu, Y., He, Z., Zhang, L., Jiang, H. & Zhang, W. Ontogeny of immunoreactive Lh and Fsh cells in relation to early ovarian differentiation and development in protogynous hermaphroditic ricefield eel *Monopterus albus*. *Biol Reprod* **86**, 93, (2012).

3 MacKay, A. B., Mhanni, A. A., McGowan, R. A. & Krone, P. H. Immunological detection of changes in genomic DNA methylation during early zebrafish

development. *Genome* **50**, 778-785 (2007).

4 Liu, Y., Song, F., Sun, J., Yu, H. & Liu, S. Y. Suture compression induced bone resorption with intensified MMP-1 and 13 expressions. *Bone* **51**, 695–703 (2012).

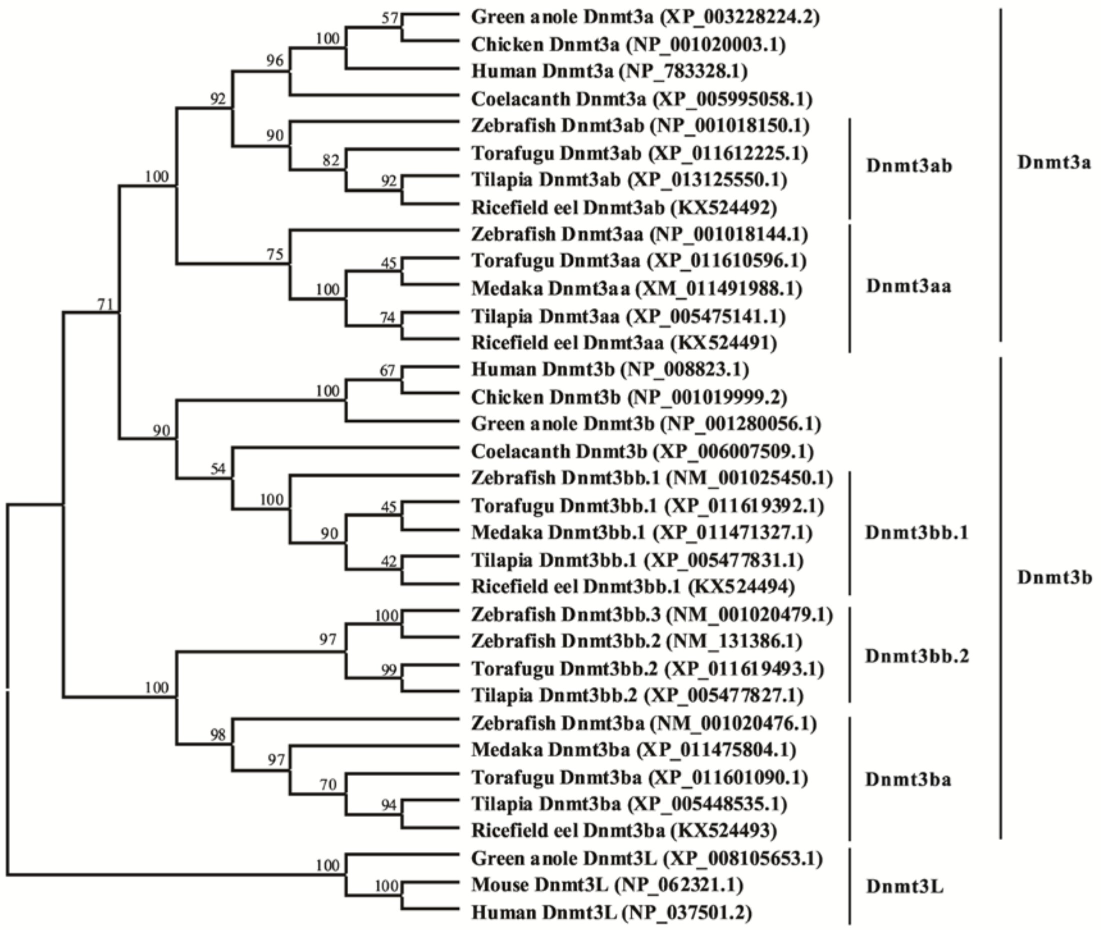

Supplementary Figure S1. Phylogenetic analysis of Dnmt3 homologues from ricefield eel and other vertebrates. The phylogenetic tree was constructed based on an alignment of deduced amino acid sequences using the Neighbor-Joining method in MEGA 6.0 software package. The percentages of replicate trees in which the associated taxa clustered together in the bootstrap test (1000 replicates) were shown next to the branches. The protein sequences were downloaded from *Entrez* (NCBI). Chicken, *Gallus gallus*; Coelacanth, *Latimeria chalumnae*; Green anole, *Anolis carolinensis*; Human, *Homo sapiens*; Medaka, *Oryzias latipes*; Mouse, *Mus musculus*; Tilapia, *Oreochromis niloticus*; Torafugu, *Takifugu rubripes*; Zebrafish, *Danio rerio*.

```
Ricefieldeel/3aa      ------------------------------------------------------------
Ricefieldeel/3ab      MPSNTVTNTNTTAFKASDFTDSPGNRMSESTIPDMDPMKEEKAAKSSASCKVGKPGRKRK 60
Zebrafish/3aa         -----------------------------------------------------------M 1
Zebrafish/3ab         ------------------------------------------------------------
Chicken/3a            ------------------------------------------------------------
Human/3a              MPAMPSSGPGDTSSSAAEREEDRKDGEEQ----EEPRGKEERQEPSTTARKVGRPGRKRK 56


Ricefieldeel/3aa      ---------MPSNCPAATDPPQTPENGTSNDVSEDGADQDS-PEEGTPASPRNKRRVGRP 50
Ricefieldeel/3ab      QFPVESCGALKDGVGNSYPGVGRPSMAQVHNGDVGGHRDRT-SDTFFLKQEKREVENGIP 119
Zebrafish/3aa         HPPTHTQRPKRDGDQQTQRHVWEKKVRLVQMSDTMDHQSAVGSQEDEPAMILPTIPIGGS 61
Zebrafish/3ab         ------------------------------------------------------------
Chicken/3a            --MVESSDTPKDTAAVPKCPPPCPEASPAEPLPNGDLEADG-AQWKGTEEGGASPKSGRP 57
Human/3a              HPPVESGDTPKDPAVISKSPSMAQDSGASELLPNGDLEKRS-EPQPEEGSPAGGQKGGAP 115


Ricefieldeel/3aa      GRKRKQLLPEMATSDPCANAPPTPPEEPEPSPSPRKKRG--------------------- 89
Ricefieldeel/3ab      RDPSSCWAEDSSQSQSLSPSQENGFLPSREEQDSEKDKDNSLTTPRKKRGRRK--LERPT 177
Zebrafish/3aa         RDKAGKKFEKKVRLVQMSDTMDHQSAVGSQEDEPAMILP--------------------- 100
Zebrafish/3ab         ----------------MNSMEDHG------EVESEPKKD-VITVP--------------- 22
Chicken/3a            EEDET----ESLADGETGRALENGRCTPKEGLDAPADEGELAPSDPQKKRGRRKLLEATE 113
Human/3a              AEGEG----AAETLPEASRAVENGCCTPKEGRGAPAEAG--------------------- 150
                                                       .

Ricefieldeel/3aa      ---RRKLEQTERNKDEPDDRNCDSPREGETGRLRRRPVPRVTFQAGDPYYISRRQREEWL 146
Ricefieldeel/3ab      KYVEHKEEDESDTVKTEGGRGRLRGGVGWEISLRQRPMPRITFQAGDPYYISKRTREEWL 237
Zebrafish/3aa         --------------TIPIGGSRDKAGKKFEKKVHLVLMS-DTMDGQSAVGSQEDEPAMIL 145
Zebrafish/3ab         -------------------------------LLFPPPMP--------------------- 30
Chicken/3a            KSKEEKEENNFDSLKMEGSRGRLRGGLGWESSLRQRPMQRHTFQAGDPYYISKRKRDEWL 173
Human/3a              ---KEQKETNIESMKMEGSRGRLRGGLGWESSLRQRPMPRLTFQAGDPYYISKRKRDEWL 207
                                                      :    :

Ricefieldeel/3aa      SRWKMEAERRAYREAEIN-----------------------------MMDDLSEADFQKE 177
Ricefieldeel/3ab      TKWKMEAEKKAKLMSAMNAIKDHKENETETQNEEVSIIKHPQPSKQQQQQQQQQPQSQPH 297
Zebrafish/3aa         PTIPQQQQKKVRLVLMSD-----------------------------TMDDQSAVGSQED 176
Zebrafish/3ab         ----------------------------------------PTPPTPPMQQQPPQPQAQHQ 50
Chicken/3a            ARWKREAEKKAKVIAVMN----------------------------VVEETPR-AEPQKE 204
Human/3a              ARWKREAEKKAKVIAGMN----------------------------AVEENQGPGESQKV 239
                                                                       :       *

Ricefieldeel/3aa      EEPASPAP------------------PPSQQHTDPASPTVAVTPEPVARGD------QAT 213
Ricefieldeel/3ab      TQSQPQYQQQSQLSLPKQQQQQHQQLPQQQQPTSPASPTVATTPEPVVIGGGEKASPKSA 357
Zebrafish/3aa         EPAMILPAIP------------------QQQHTDPASPTVATTPEPPPVACGEKVLTGIA 218
```

```
Zebrafish/3ab       TNLKPQPS-------PQQQPPPFQQQ-QQQQPTDPASPTVATTPEPVSIGDGDKTSPKS- 101
Chicken/3a          EEASPPAS---------------------QQPTDPASPNVATTPEPVVADAVDKNTSKSA 243
Human/3a            EEASPPAV---------------------QQPTDPASPTVATTPEPVGSDAGDKNATKAG 278
                                                 ** *.****.**.****

Ricefieldeel/3aa    PSEIEYQDGRGFGIGTLVFGKLRGFSWWPGRIVSWWMSGRSRAADGTRWVMWFGDGKFSV 273
Ricefieldeel/3ab    DTESEYEDSRGFGIGELVWGKLRGFSWWPGRIVSWWVTGRSRAAEGTRWVMWFGDGKFSV 417
Zebrafish/3aa       PIHQEYQDGLGYGIGELVWGKLRGFSWWPGRIVSCWITNRGPAAEGTRWVMWFGDGKLSM 278
Zebrafish/3ab       -TESEYEDGRGFGINELVWGKLRGYSWWPGRVVSWRMTRRSPAAEGTRWVTWFGDGKFSV 160
Chicken/3a          DDEPEYEDGRGLGIGELVWGKLRGFSWWPGRIVSWWMTGRSRAAEGTRWVMWFGDGKFSV 303
Human/3a            DDEPEYEDGRGFGIGELVWGKLRGFSWWPGRIVSWWMTGRSRAAEGTRWVMWFGDGKFSV 338
                      . **:*. * **. **:*****:******:**  :: *. **:***** ******:*:

                    PWWP domain
Ricefieldeel/3aa    VCVEKLMPLSSFSSAFHQPTYNKQSMYRKAIFEALQVASVRAGRPVPSCDASDDADG--- 330
Ricefieldeel/3ab    VCVEKLMPLSSFNNAFHQPTYNKQPMYKKAIYEVLQVASSRAGKAFMACPDSDETETSKS 477
Zebrafish/3aa       MCVEKLLPLSAFSKTFHQPTYNKQPVYRKAIFQVLLTGGLRAGKPFPHCEYTNYTPEGKM 338
Zebrafish/3ab       VCVEKLLPLSSFHNAFHQPTYNKQPMYKKAIFEVLQVASTRAGKTFLSCPASDDTDSSKL 220
Chicken/3a          VCVEKLLPLSSFSSAFHQATYNKQPMYRKAIYEVLQVASSRAGKIFPACPENDETDTSKV 363
Human/3a            VCVEKLMPLSSFCSAFHQATYNKQPMYRKAIYEVLQVASSRAGKLFPVCHDSDESDTAKA 398
                    :*****:***:* .:***.*****.:*:***::.* ... ***: .  *  .: :

Ricefieldeel/3aa    --VEIQTRQMIEWAMTGFLPSGPQSLDPPEEEQNPYKEVYPEMWSEPE-AAYTPPP-AKK 386
Ricefieldeel/3ab    --VEMLNKQMIDWAMAGFHPTGPKGLEPPEEERSPYKEVYPEIWVEPEAAAYTPPP-AKK 534
Zebrafish/3aa       EENETQAQQMIKWASTGFLPSGPKGLEPPPAERNPYTEVYPEMW-EPEAASYTAPPPAKK 397
Zebrafish/3ab       --VDVENKKMIEWAMSGFQPTGPKGLDPPESE--PYEMCE---WVEPEAASYLPPP-AKK 272
Chicken/3a          --VEIQNKQMIEWALGGFQPSGPKGLEPPEEERNPYKEVYTEMWVEPEAAAYAPPPPAKK 421
Human/3a            --VEVQNKPMIEWALGGFQPSGPKGLEPPEEEKNPYKEVYTDMWVEPEAAAYAPPPPAKK 456
                       :   : **.**  ** *:**:.*:**  *  **       * *** *:* .** ***

Ricefieldeel/3aa    PRKNSAE-KAKIREVIDEGTRERLIYEIKKKTRNIEDICISCGSLNVSLEHPLFVGAMCQ 445
Ricefieldeel/3ab    PRKSTAE-KPKVKDIIDERTRERLVYEVRQKCRSIEDICISCGSLNVSLEHPLFAGGMCQ 593
Zebrafish/3aa       PRKNSVE-KTKIKEIIDEGTRERLMLEVKQKCRNIEDICISCGSLNVTLEHPLFVGAMCQ 456
Zebrafish/3ab       PRKSTVTEKPKVKEMIDERTRERLVFDVRQKCRNIEDICISCGSLYVSLEHPLFIGGMCQ 332
Chicken/3a          PRKSTTE-KPKVKEIIDERTRERLVYEVRQKCRNIEDICISCGSLNVTLEHPLFIGGMCQ 480
Human/3a            PRKSTAE-KPKVKEIIDERTRERLVYEVRQKCRNIEDICISCGSLNVTLEHPLFVGGMCQ 515
                    ***.:.  *.*::::*** *****: ::::* *.*********** *:****** *.***

                    PHD-like Zinc finger domain
Ricefieldeel/3aa    GCKNSFLECAYQYDDDGYQSYCTICCGGREVLMCGNNNCCRCFCVECVDLLVGAGSAAAA 505
Ricefieldeel/3ab    SCKNCFLECAYQYDDDGYQSYCTICCGGREVLMCGNNNCCRCFCVECVDLLVGQGAAHAA 653
Zebrafish/3aa       SCKNSFLECAYQYDEDGYQSFCTICCGGRQVLMCGNNNCCRCYCVECVDLLVGTGCAQAA 516
Zebrafish/3ab       SCTNCFLECAYQYDDDGYQSYCTICCGGREVLMCGNNNCCRCFCVECVDLLVGPGAAQAA 392
Chicken/3a          NCKNCFLECAYQYDDDGYQSYCTICCGGREVLMCGNNNCCRCFCVECVDLLVGPGAAQAA 540
```

```
Human/3a               NCKNCFLECAYQYDDDGYQSYCTICCGGREVLMCGNNNCCRCFCVECVDLLVGPGAAQAA 575
                       .*.*.*********:*****:********:************:********** *.* **

Ricefieldeel/3aa       IKEDPWNCYMCGPRSTYGLLRRRDDWPCRLQHFFVNNHEQEFEPAKLYPPVVAEKRQPIR 565
Ricefieldeel/3ab       IKEDPWNCYMCGQKGVFGLLERRTDWPSRLQHFFANNHDQDFDPPKLYAPVMVEKRKPIR 713
Zebrafish/3aa          ISEDPWNCYMCCSRSVFGLLRRRDDWTSRLQLFFANNHDQDFEPPALYSPVAPEKRQPIR 576
Zebrafish/3ab          IKEDPWNCYMCGLKTQYGLLERRADWPCRLQHFFANNHDQEFEPPRLYPPVLAENRKPIR 452
Chicken/3a             IKEDPWNCYMCGHKGVYGLLRRREDWPSRLQMFFANNHDQEFDPPKVYPPVPAEKRKPIR 600
Human/3a               IKEDPWNCYMCGHKGTYGLLRRREDWPSRLQMFFANNHDQEFDPPKVYPPVPAEKRKPIR 635
                       *.*********  :  :***.** **..*** **.***:*:*:*. :*.**  *:*:***
```

C-terminal catalytic domain

```
Ricefieldeel/3aa       VLSLFDGIATGLLVLKDLGIQVDKYVASEVCEDSITVGIVRHQGRIMYVGDVRNVTRKHI 625
Ricefieldeel/3ab       VLSLFDGIATGLLVLKELGIQVDRYVASEVCEDSITVGIVRHQGRIMYVGDVRTVTRKHI 773
Zebrafish/3aa          VLSLFDGIATGLLVLKDLGIQVDRYVASEVCEDSITVGMVRHFERITYVGDIRNITRKHI 636
Zebrafish/3ab          VLSLFDGIATGLLVLRELGIQVERYVASEVCEDSITVGIVRHQGRIMYVGDVRQLTRKNI 512
Chicken/3a             VLSLFDGIATGLLVLKDLGIQVDRYIASEVCEDSITVGMVRHQGKIMYVGDVRNVTQKHI 660
Human/3a               VLSLFDGIATGLLVLKDLGIQVDRYIASEVCEDSITVGMVRHQGKIMYVGDVRSVTQKHI 695
                       ***************::*****::*:************:***   :* ****:* :*:*:*

Ricefieldeel/3aa       EEWGPFDLVIGGSPCNDLSIVNPARKGLYEGTGRLFFEFYRLLHEARPKPDDERPFFWLF 685
Ricefieldeel/3ab       QEWGPFDLVIGGSPCNDLSIVNPARKGLYEGTGRLFFEFYRLLHEARPKNGEDRPFFWLF 833
Zebrafish/3aa          QEWGPFDLVIGGSPCNDLSIVNPARKGLFEGTGRLFFEFYRLLHEARPKEGDDRPFFWLF 696
Zebrafish/3ab          QEWGPFDLVIGGSPCNDLSIVNPARKGLYEGTGRLFFEFYRLLHEARPKEGDTRPFFWLF 572
Chicken/3a             QEWGPFDLVIGGSPCNDLSIVNPARKGLYEGTGRLFFEFYRLLHEARPKEGDDRPFFWLF 720
Human/3a               QEWGPFDLVIGGSPCNDLSIVNPARKGLYEGTGRLFFEFYRLLHDARPKEGDDRPFFWLF 755
                       :***************************:***************:**** .: *******

Ricefieldeel/3aa       ENVVAMGVSDKRDISRFLECNPVMIDAKEVSAAHRARYFWGNLPGMSRPLTPMANDKLDL 745
Ricefieldeel/3ab       ENVVAMGVSDKRDISRFLECNPVIIDAKEVSAAHRARYFWGNLPGMNRPLSAMCTDRLDL 893
Zebrafish/3aa          ENVVAMGVSDKKDISRFLECNPVMIDAKEVSAAHRARYFWGNLPGMNRPLTAMVNDKLDL 756
Zebrafish/3ab          ENVVAMGVSDKRDISRFLECNPVMIDAKEVSAAHRARYFWGNLPGMNRPMSAMCTDKLDL 632
Chicken/3a             ENVVAMGVSDKRDISRFLESNPVMIDAKEVSAAHRARYFWGNLPGMNRPLASTVNDKLEL 780
Human/3a               ENVVAMGVSDKRDISRFLESNPVMIDAKEVSAAHRARYFWGNLPGMNRPLASTVNDKLEL 815
                       ***********:*******.***:*********************.**::.   .*:*:*

Ricefieldeel/3aa       QDCLEHGRTAKFEKLRTITTRSNSVKQGKDEHFPVYMDNKEDILWCTEMERVFGFPVHYT 805
Ricefieldeel/3ab       QDCLEHGRTAKFDKVRTITTRSNSIKQGKDQHFPVYMNEKEDILWCTEMERVFGFPVHYT 953
Zebrafish/3aa          QDCLEHGRTAKFNKVRTITTRSNSIKQGKDQHYPVYMNNKEDILWCTEMERVFGFPVHYT 816
Zebrafish/3ab          QDCLEHGRTAKFGKVRTITTRSNSIKQGKDQHFPVFMNDKEDILWCTEMERVFGFPVHYT 692
Chicken/3a             QECLEHGRIAKFSKVRTITTRSNSIKQGKDQHFPVFMNEKEDILWCTEMERVFGFPVHYT 840
Human/3a               QECLEHGRIAKFSKVRTITTRSNSIKQGKDQHFPVFMNEKEDILWCTEMERVFGFPVHYT 875
                       *:****** *** *:*********:*****:*:**:*:*:********************
```

```
Ricefieldeel/3aa      DVSNMSRLARQRLLGRSWSVPVIRHLFAPLKEYFACN-- 842
Ricefieldeel/3ab      DVSNMSRLARQRLLGRSWSVPVIRHLFAPLKDYFACV-- 990
Zebrafish/3aa         DVSNMSRLARQRLLGRSWSVPVIRHLFAPLKEYFAC--- 852
Zebrafish/3ab         DVSNMSRLARQRLLGRSWSVPVIRHLFAPLKEYFACVGT 731
Chicken/3a            DVSNMSRLARQRLLGRSWSVPVIRHLFAPLKEYFACV-- 877
Human/3a              DVSNMSRLARQRLLGRSWSVPVIRHLFAPLKEYFACV-- 912
                      *******************************:****
```

Supplementary Figure S2. The alignment of amino acid sequences of Dnmt3a homologues from ricefield eel and other representative vertebrates. The sequence alignment was performed with the software Clustal X 1.83. The conserved PWWP domain, PHD-like Zn finger domain and C-terminal catalytic domain are marked in gray. The protein sequences were downloaded from *Entrez* (NCBI). For details, please refer to Supplementary Figure S1.

```
Ricefieldeel/3ba      MASTAVVTPDLPNDKPNHFHLVSWMNNLLQTDFKDVREMCSGACHCQIMDWIFPGSIDMT 60
Ricefieldeel/3bb.1    ------------------------------------------------------------
Zebrafish/3ba         MATNVSLEPNNPDDKCSRYEVLGWINETLQTNFTQVEQCRSGACFCQLIDLLFPGTINLK 60
Zebrafish/3bb.1       ------------------------------------------------------------
Chicken/3b            ------------------------------------------------------------
Human/3b              ------------------------------------------------------------

                      ——— calponin-homology (CH) domain ——→
Ricefieldeel/3ba      QVKFDAQGEDDFKHNFSLLHEAFSKRGIMRTIPVEELIKGDFKSNVQIPKWFKAFYKANV 120
Ricefieldeel/3bb.1    ------------------------------------------------------------
Zebrafish/3ba         KVKFESQKRSDFMQNYSLLQAAFRDLEVTEPVPVNELLSGKFRPNFTYLKWFKKFFYANV 120
Zebrafish/3bb.1       ------------------------------------------------------------
Chicken/3b            ------------------------------------------------------------
Human/3b              ------------------------------------------------------------

Ricefieldeel/3ba      KCE-AYDPVKARDSRVISP---VVGSPLSHKWSSKLESDMEENDTETT---KDFLYTEKW 173
Ricefieldeel/3bb.1    ------------------------------------------------------------
Zebrafish/3ba         KQERVYNAFEARDGQEIVPVDDVMKSPKALKSSYESGRAGEESDMEINGGRRSATYDPKW 180
Zebrafish/3bb.1       ------------------------------------------------------------
Chicken/3b            ------------------------------------------------------------
Human/3b              ------------------------------------------------------------

Ricefieldeel/3ba      KDIFDWAERSTLGEQYTYCRCCLKNLTTFHKGLIDLRRHGETATHKKRATTFISADPQSQ 233
Ricefieldeel/3bb.1    ------------------------------------------------------------
Zebrafish/3ba         QRNLKWIRASDMGDNYAYCTTCDYNIILLAG-FHDLKRHQLTQNHMKHETGRTNLPGRKQ 239
Zebrafish/3bb.1       ------------------------------------------------------------
Chicken/3b            ------------------------------------------------------------
Human/3b              ------------------------------------------------------------

Ricefieldeel/3ba      LSEPLPCSDAALRFIHNHCYTGS-AKGEEVSKHFARSKLGPRYPKDIASVCQHTPYCVYI 292
Ricefieldeel/3bb.1    ------------------------------------------------------------
Zebrafish/3ba         IEESISCSETMLLFIQSHCLSSLPSRINRVSQRTARCILGLKYPNDIVSACKLNPYCIYI 299
Zebrafish/3bb.1       ------------------------------------------------------------
Chicken/3b            ------------------------------------------------------------
Human/3b              ------------------------------------------------------------

Ricefieldeel/3ba      Y----IGVTVGKDDTVSVVLVGFFDVEASRYCIRFLDALQAGEGAGDQTEAAAVVETLKK 348
Ricefieldeel/3bb.1    ------------------------------------------------------------
Zebrafish/3ba         YGQVPLDVKTGDKTNCHVVLAGFFEEKQARYCIRFLDVFQPEDSAS--SVSGGLFSILKK 357
Zebrafish/3bb.1       ------------------------------------------------------------
Chicken/3b            ------------------------------------------------------------
```

```
Human/3b                ------------------------------------------------------------

Ricefieldeel/3ba        FELPRDNLVAVYCDGNGAVSEQICSQLRELSPNIVALGELYTIADAACSAGVKWLSSHTQ 408
Ricefieldeel/3bb.1      ------------------------------------------------------------
Zebrafish/3ba           FEIPASNMVAVYINDHELTSESVVSQIRELNPQVIDLGGLYSIPDTACSAGLQTHSVQVE 417
Zebrafish/3bb.1         ------------------------------------------------------------
Chicken/3b              ------------------------------------------------------------
Human/3b                ------------------------------------------------------------

Ricefieldeel/3ba        ELIALIHAHNSSCTKKNDNLDTLFGSDISVDSQSFHLNTSCLKFYLFITKILGMWTDLML 468
Ricefieldeel/3bb.1      --------------------------------------------------------MVMF 4
Zebrafish/3ba           ELIANIYRHFSTGSTSNDNLKMLFAGIDGLKVHSNPLSNS-EEFCVLVKRIHEMWSDLVS 476
Zebrafish/3bb.1         -----------------------------------------------------------M 1
Chicken/3b              ----------------------------------------------------------MS 2
Human/3b                ----------------------------------------------------------MK 2

Ricefieldeel/3ba        YFKSCEKNDDKAKLIFSKLQDPKVRATFMFLEQALKPLHSFQKHLQTQEEAPRADMLLIL 528
Ricefieldeel/3bb.1      EKESGQPLDQSTVTAMPSNKYSAVIMEESNNMTATAAVNGDTPPGEGLSENDSGVELTSE 64
Zebrafish/3ba           YFSSCDENNDNVKQICSQLENPKIRLTLMFLDQALGPLRAFGQHLQQSKSSVRADLVEIL 536
Zebrafish/3bb.1         RKEE---IKKSTEIVMPSNKYPS---AESDKMTATAAMNRDTSVGDGLSENDSGLEMTSE 55
Chicken/3b              MVTSLERLEQRLAVMVAAQETSQAWSSLGE--EGPAAMKKEKSHGRDEADCRAELILF-D 59
Human/3b                GDTRHLNGEEDAGGREDSILVNGACSDQSS--DSPPILEAIRTP--EIRGRRSSSRLS-K 57
                                ..                       .   :.                 :

Ricefieldeel/3ba        EEASSLLCTYTSYFLHPQAAARFLKEHDAQILKNKKFHLSSSELNLGGKAVEDFLNESDS 588
Ricefieldeel/3bb.1      NSPLTAAEPPSPFSLKQNGGAASPQDGNQCSRGSRKRSRKRAE----------------- 107
Zebrafish/3ba           REASGLLRSYASSFLRPQAVIRYLKEQDPAILDNEAFCLPAAELSLGG-VLEDFISAREK 595
Zebrafish/3bb.1         NSPLTPAEPPSPFCPKQNGGAASPADESVNSIR-RKRSRKRSDT---------------- 98
Chicken/3b              GDCTDPTKDTAALLLEASGKPGAPDAGSLGLAPLKNKRVSKDLS---------------- 103
Human/3b                REVSSLLSYTQDLTGDGDGEDGDGSD-----TPVMPKLFRETRT---------------- 96
                         .               ..

Ricefieldeel/3ba        --AEVLPLLKQEVLSFYIALTSCIAEGLPFSDGVLRSIAQLLNPQSRLKVTGKAVGELGT 646
Ricefieldeel/3bb.1      ----------EEGSTWDS----CSEEKSSGTS--QLGLRQRP-RPRTIFQAGLTAQTHTK 150
Zebrafish/3ba           ELADFLSTFYNECLAIYKTLTTSIAASLPLSDSVLRAISQLLSPAGRLKVTGKNIVDLAV 655
Zebrafish/3bb.1         ----------EEDSAWDSS---YSEEKAEVGSGCETGLRQRP-RPRTIFQAGLTAHSKPR 144
Chicken/3b              ----------KEELSWPLTLMEPQEVRPRGSAGWESSLRQKP-PVRLIFQAGQTHHEMQI 152
Human/3b                ----------RS---------ESPAVRTRNNNSVSSRERHRPSPRSTRGRQGRNHVDESP 137
                                  ..                           :           *
Ricefieldeel/3ba        KLGICSSPKETNQLTSEFLEYQLAEEGESEEGEKDKSAEVSLDRHWASVLKDTEPTSVFR 706
Ricefieldeel/3bb.1      SR-RQNRK-----------------------------------------QEHSIIVYGGG 168
Zebrafish/3ba           RFGFCSKPEDSAKLNDEFLEYQLAEE-------ENLSSTHSIERYWCTVLKTFPPTSVFK 708
Zebrafish/3bb.1         SRERGHSK-----------------------------------------EDHSDLVAS-- 161
Chicken/3b              KESSSVEA-----------------------------------------LRELPAPLRSS 171
```

```
Human/3b              VEFPATRS-----------------------------------------LRRRATASAGT 156

Ricefieldeel/3ba      KLVLTLLSLPFPPLDSHQVFSQALENRDSALFSDSEALTKSKYDVMSDSNLSDSSTSKDS 766
Ricefieldeel/3bb.1    PRAVTAVSSGVPETPRLELME--QDSKDSAQSSSTSSSS---EIQP-------------- 209
Zebrafish/3ba         RLVLCLLVLPSPSLDATKIFAQAIENGDADQLDDSSSESDTDMTKELDSNDDNSLD---- 764
Zebrafish/3bb.1       ----------VPEGPALELME--QDSKDSAQSSTTSTSTTETASQP-------------- 195
Chicken/3b            RR-----RTAVPVLTTIDLTE--EDSRDSSQSSSTLSGSSSQEGQNGST----------- 213
Human/3b              PW-----PSPPSSYLTIDLTDDTEDTHGTPQSSSTPYARLAQDSQQGGM----------- 200
                                .     .:     :. .:   . :

Ricefieldeel/3ba      PVCTSEPNNLNVTLKLCEVRLTKISRPGNENDHVPGENGAVCSEGTGRGSFGWESSLRKK 826
Ricefieldeel/3bb.1    ------------------------------------------------------------
Zebrafish/3ba         ----------NSELQISPIKNGIMKKSRRSTSEVNLKNDGAKNDGT-------------- 800
Zebrafish/3bb.1       ------------------------------------------------------------
Chicken/3b            ------------------------------------------------------------
Human/3b              ------------------------------------------------------------

Ricefieldeel/3ba      PQARTLFQAGASTWSKPVVLEKDTKNDPKPQDEGVQAEESSPSNNSTPRGRRKHAYRDGK 886
Ricefieldeel/3bb.1    ------------------------------------------------------EYNDNK 215
Zebrafish/3ba         ------------------LTSNTLKEVKN--------DLSNSTPSPRRGKRDQAYNDGK 833
Zebrafish/3bb.1       ------------------------------------------------------EYKDNK 201
Chicken/3b            -------------------------------------ELVAEEPESRDAGIALEYQDGK 235
Human/3b              -------------------------------------ESPQVEADSGD-GDSSEYQDGK 221
                                                                         *.*.*

                      <------------------------------ PWWP domain ---------
Ricefieldeel/3ba      GFLTGELVWGKVRGFSWWPGMVMPWKS---KSAPPGMRRVEWFGDGMFSEICTDGLLGFN 943
Ricefieldeel/3bb.1    GFGIGELVWGKIKGFSWWPGIVVTWRATGKRQANHGMRWLQWFGDGKFSEVSADKLESIT 275
Zebrafish/3ba         GFAVGELVWGKVKDFSLWPGLVVPWKG---RIVPVSMRRVEWFGDGMFSEIHTDGLLPFG 890
Zebrafish/3bb.1       GFGIGELVWGKIKGFSWWPGMVVTWRATGRRQASHGMRWLQWFGDGKFSEVSADKLDSIT 261
Chicken/3b            EFGIGELVWGKIKGFSWWPAIVVSYRATSKRQAVSGMRWVQWFGDGKFSEVSADKLVGLM 295
Human/3b              EFGIGDLVWGKIKGFSWWPAMVVSWKATSKRQAMSGMRWVQWFGDGKFSEVSADKLVALG 281
                       *  *:*****::.** **.:*:.::.   : .  .** ::***** ***: :* *  :

                      ---------------------->
Ricefieldeel/3ba      AFTKCFCKNSFASLPIYKDAIYQIIELAGERCGKSFSEAGG-NREKELKLMVDWALEGFL 1002
Ricefieldeel/3bb.1    AFPKFFSQASYTKLASYRRAIFQALEMASSRAEKTFPSCESDNPEDQVKPMLDWANGGFL 335
Zebrafish/3ba         AFSKNFCSKSYEGLPTYKNAIYQILELAAERSGKLFPPSEK-KGE-EVKAMMDWAFGGFQ 948
Zebrafish/3bb.1       AFPKFFNQSSYTKLASYRRAIFQALEVASLRAEKTFPPSEADSLEEQVKPMLDWAHGGFL 321
Chicken/3b            AFRQHFNTATFNKLVSYRRAIYHALEVARSRSGKTFTSAPGESLEEQLKPMIDWALTGFK 355
Human/3b              LFSQHFNLATFNKLVSYRKAMYHALEKARVRAGKTFPSSPGDSLEDQLKPMLEWAHGGFK 341
                       * : *   ::  *  *: *::: :* *  *. * *. .   . * ::* *::**  **

Ricefieldeel/3ba      PTGPEGFIP------------------------PDSAAHHESTDSALSDYQPPAKK--- 1034
Ricefieldeel/3bb.1    PKGEEGLKPTY----------------NTN--SNPLDHQ---VLDLSLPEYFPTAKRPKD 374
```

```
Zebrafish/3ba          PMGADGFLPSA------------------------DSSASNKTESDSSVSDYQPPAKR--- 982
Zebrafish/3bb.1        PKGQEGLKPKE-----------------NAEYCVFPLASESSTLLESSPPEFPPSAKRARL 365
Chicken/3b             PLGVKGLQP---------------------PKSSENGALRNGTEEVLCLEHCPPTKRLKS 394
Human/3b               PTGIEGLKPNNTQPVVNKSKVRRAGSRKLESRKYENKTRRRTADDSATSDYCPAPKRLKT 401
                       * * .*: *                                :    :. *..*:

Ricefieldeel/3ba       --KYVLKNK-TNASCSRESIIEKVKEKGKTIEDFCLSCGSSELDVPHPLFEGGLCLKCKG 1091
Ricefieldeel/3bb.1     S--LCKNKAPPEESCSRERMVNEVLKNKKNIEEFCLSCGNMRVATFHPLFEGGLCQTCKD 432
Zebrafish/3ba          --KYVFKNRPSTQECNRDQMVQEVTSKGRKIEDFCLSCGSSNTETFHPLFKGSLCIKCKE 1040
Zebrafish/3bb.1        P--LNKAKPGIEEVYSREQMVNEVLKNHRSIEEFCLSCGKTRVATFHPLFEGGLCLTCKD 423
Chicken/3b             NPCNSSKEQRMEEDQTREQMVSEVTNNSGSLEDSCLSCGRRNPATFHPLFEGGLCHTCRD 454
Human/3b               N-CYNNGKDRGDEDQSREQMASDVANNKSSLEDGCLSCGRKNPVSFHPLFEGGLCQTCRD 460
                           :       .*: : ..* .:  .:*: *****  .    ****:*.** .*:

                       PHD-like Zinc finger domain
Ricefieldeel/3ba       NFTETLYRYDEDGYQSYCTVCCGGLEVILCGNASCCRCFCKDCLDILVSPGTFDKLKDVD 1151
Ricefieldeel/3bb.1     VYLEMSYMYDDDGYQSYCTICCGGREVLLCGNANCCRCFCVDCLDILVSPGASNSARYVD 492
Zebrafish/3ba          NFTETLYRYDDDGYQSYCTVCCAGLEVILCGNASCCRCFCKDCLNVLVGPGTFDKLKEVD 1100
Zebrafish/3bb.1        AYLENSYMYDDDGYQSYCTVCCGGREMLLCGNANCCRCICVDCLDILVGAGAANSARNLD 483
Chicken/3b             RFLELFYMYDEDGYQSYCTVCCEGKELLLCSNASCCRCFCVECLEVLVGRGSSAKAKEQE 514
Human/3b               RFLELFYMYDDDGYQSYCTVCCEGRELLLCSNTSCCRCFCVECLEVLVGTGTAAEAKLQE 520
                        : *  * **:********:** * *::**.*:.****:* :**::**. *:  . :  :

Ricefieldeel/3ba       PWSCYMCQPSQCAGNLKLRPDWSVKVQDLFVNNSAMEFEPHRVYPSIPADRRRPIKVLSL 1211
Ricefieldeel/3bb.1     PWRCYMCQPLLQYGAIKRRHDWSLKLQEFFANDNGQEFEKPKIYPAVPAEQRQPIRVLSL 552
Zebrafish/3ba          PWSCYVCLPSKCYGVLKLRTDWSVRVQEFFANNSAFEFEPHRVYPSIPAHKRRPIRVLSL 1160
Zebrafish/3bb.1        PWRCYMCQPLQQYGVLKKRHDWSLKLQEFFVNDSGQEFESPKIYPAVPAEQRRPIRVLSL 543
Chicken/3b             PWNCYMCQPQRSYGVLQRRQDWSSRLQDFFTSDKGQEYDAPKIYPAVPPAKRRPIRVLSL 574
Human/3b               PWSCYMCLPQRCHGVLRRRKDWNVRLQAFFTSDTGLEYEAPKLYPAIPAARRRPIRVLSL 580
                       ** **:* *    * :: * **. ::* :*..:.. *::  ::**::*. :*:**:****

Ricefieldeel/3ba       FDGIATGYLVLKDLGFKIERYIASEICEDSIAVGMVKHEGKIEYVNDVRTITRKHLAEWG 1271
Ricefieldeel/3bb.1     FDGIVTGYLVLRDLGFKVGQYVASEVCEDSISVGVVRHEGKIQYVHDVRNITKKNIQEWG 612
Zebrafish/3ba          FDGIATGYLVLKDLGFKLERYIASEICEDSIAVGMVKHEGKIEYVKDVRTITRKHLAEWG 1220
Zebrafish/3bb.1        FDGIATGYLVLRDLGFKVDLYIASEVCEDSISVGAVRHEGKIQYVHDVRNITRKNIAEWG 603
Chicken/3b             FDGIATGYLVLKDLGIQVEKYIASEICEDPLAVGTVRHEGNITYVHDVRNITKRNIEEWG 634
Human/3b               FDGIATGYLVLKELGIKVGKYVASEVCEESIAVGTVKHEGNIKYVNDVRNITKKNIEEWG 640
                       ****.******::**:::   *:***:**:.::** *:***:* **:***.**:::: ***

Ricefieldeel/3ba       PFDLLIGGSPCNDLSMVNPLRKGLF---------------------------------- 1296
Ricefieldeel/3bb.1     PFDLVIGGSPCNDLSIVNPARKGLY---------------------------------- 637
Zebrafish/3ba          PFDLLIGGSPCNDLSMVNPARKGLF---------------------------------- 1245
Zebrafish/3bb.1        PFDMVIGGSPCNDLSIVNPARKGLY---------------------------------- 628
Chicken/3b             PFDLVIGGSPCNDLSLASPPRKAPYGEAAWPHGAGSPQAAWGRRRSCAVAPSGDTCQALS 694
```

```
Human/3b               PFDLVIGGSPCNDLSNVNPARKGLY----------------------------------- 665
                       ***::********** ..* **. :

Ricefieldeel/3ba       --------------------------------EGTGRLFFEFYRILTLLKPKEGDDRPFF 1324
Ricefieldeel/3bb.1     --------------------------------EGTGRLFFEFYRQLSEAKPKEGENRPFF 665
Zebrafish/3ba          --------------------------------EGTGRLFFEYYRMLTMMRPKEDDDRPFF 1273
Zebrafish/3bb.1        --------------------------------EGTGRLFFEFYRLLSEAKPKEGEDRPFF 656
Chicken/3b             PPGPPEPVPTPQPNLRGRGRSPPNTPAGSPRTEGTGRLFFEFYHLLNYARPKAGEERPFF 754
Human/3b               --------------------------------EGTGRLFFEFYHLLNYSRPKEGDDRPFF 693
                                                       *********:*: *.   :** .::****
```

C-terminal catalytic domain

```
Ricefieldeel/3ba       WLFENVVFMSANDKSDICRFLECNPILIDAVKVSPAHRARYFWGNLPGMNRPLSTFLDDK 1384
Ricefieldeel/3bb.1     WMFENVVAMGVNDKRDISRFLECNPVMIDAIEVSAAHRARYFWGNLPGMNRPLCASGMDK 725
Zebrafish/3ba          WLFENVVAMSAHDKADICRFLECNPVMIDAVKVSPAHRARYFWGNLPGMNRPVATSLTDN 1333
Zebrafish/3bb.1        WMFENVVAMSVNDKRDISRFLECNPVMIDAIEVSAAHRARYFWGNLPGMKRPLCASGMDK 716
Chicken/3b             WMFENVVAMRVNDKRDISRFLECNPVMIDAIKISAAHRARYFWGNLPGMN---------- 804
Human/3b               WMFENVVAMKVGDKRDISRFLECNPVMIDAIKVSAAHRARYFWGNLPGMNRPVIASKNDK 753
                       *:***** * .  ** **.*******::***:::*.**************:

Ricefieldeel/3ba       VALQDCLEVGRMAKFDKVRTITTKSNSIRQGKMGPLPVSMNGKEDYLWCTEMEQVFGFPK 1444
Ricefieldeel/3bb.1     LELQDCLEHGRVAKFGKVRTITTRSNSIKQGKDQHFPVLMNGKEDILWCTELERIFGFPV 785
Zebrafish/3ba          VDLQDCLESGRTAMFSKVRTITTKSNSIKQGKTGPLPVTMNGKEDYLWCTEMEKIFGFPK 1393
Zebrafish/3bb.1        LELQDCLEHGRVAKFGKVRTITTRSNSIKQGKDQHFPVMMNGKEDILWCTELERIFGFPV 776
Chicken/3b             -----------------------------------------------------RIFGFPL 811
Human/3b               LELQDCLEYNRIAKLKKVQTITTKSNSIKQGKNQLFPVVMNGKEDVLWCTELERIFGFPV 813
                                                                            ::****

Ricefieldeel/3ba       HYTDVNNMGRMQRQKVLGRSWSVPVIRHLFAPLKDYFECE 1484
Ricefieldeel/3bb.1     HYTDVSNMGRGARQKLLGRSWSVPVIRHLFAPLKDYFACE 825
Zebrafish/3ba          HYTDVNNMGRGQRQKVLGRSWSVPVIRHLFAPLKDYFACE 1433
Zebrafish/3bb.1        HYTDVSNMGRGARQKLLGRSWSVPVIRHLFAPLKDYFACE 816
Chicken/3b             HCTDVSNISRGARQKLLGRSWSIPVIQHLFSPLKDYFACE 851
Human/3b               HYTDVSNMGRGARQKLLGRSWSVPVIRHLFAPLKDYFACE 853
                       * ***.*:.*  ***:******:***:***:****** **
```

Supplementary Figure S3. The alignment of amino acid sequences of Dnmt3b homologues from ricefield eel and other representative vertebrates. The sequence alignment was performed with the software Clustal X 1.83. The conserved calponin-homology (CH) domain, PWWP domain, PHD-like Zn finger domain and C-terminal catalytic domain are marked in gray. The protein sequences were downloaded from *Entrez* (NCBI). For details, please refer to Supplementary Figure S1.

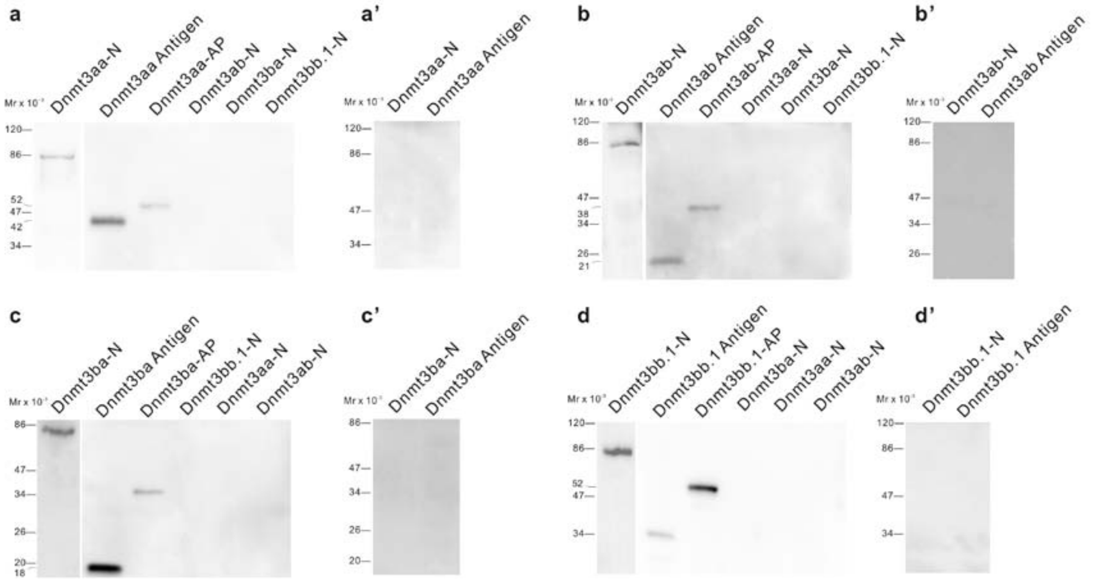

Supplementary Figure S4. The specificity of anti-ricefield eel Dnmt3aa, Dnmt3ab, Dnmt3ba or Dnmt3bb.1 antiserum as determined by Western blot analysis. The proteins were separated on 8% SDS-PAGE gels, transferred to polvinylidene fluoride membranes, and then immunoreacted with: (a) the mouse anti-ricefield eel Dnmt3aa antiserum (1:1000); (b') anti-Dnmt3aa antiserum pre-adsorbed by excessive Dnmt3aa-N; (b) the mouse anti-ricefield eel Dnmt3ab antiserum (1:1000); (b') anti- nmt3ab antiserum pre-adsorbed by excessive Dnmt3ab-N; (c) the mouse anti-ricefield eel Dnmt3ba antiserum (1:1000); (c') anti-Dnmt3ba antiserum pre-adsorbed by excessive Dnmt3ba-N; (d) the mouse anti-ricefield eel Dnmt3bb.1 antiserum (1:1000); (d') anti-Dnmt3bb.1 antiserum pre-adsorbed by excessive Dnmt3bb.1-N. Dnmt3aa-N, Dnmt3ab-N, Dnmt3ba-N, and Dnmt3bb.1-N, the recombinant N-terminal portions of corresponding Dnmt3 encompassing the antigen regions; Dnmt3aa antigen, Dnmt3ab antigen, Dnmt3ba antigen, and Dnmt3bb.1 antigen, the recombinant polypeptide fragments of corresponding Dnmt3 homologues used to generate antisera; Dnmt3aa-AP, Dnmt3ab-AP, Dnmt3ba-AP, and Dnmt3bb.1-AP, the same target polypeptides as the antigens of corresponding Dnmt3 homologues prepared with another expression vector different from the one used for the antigens. The secondary antibody was 1:5000 diluted horseradish peroxidase (HRP)-conjugated goat anti-mouse IgG (H+L) (115-035-003, Jackson ImmunoResearch Laboratories, Inc.). The blots were visualized using the BeyoECL Plus kit (Beyotime).

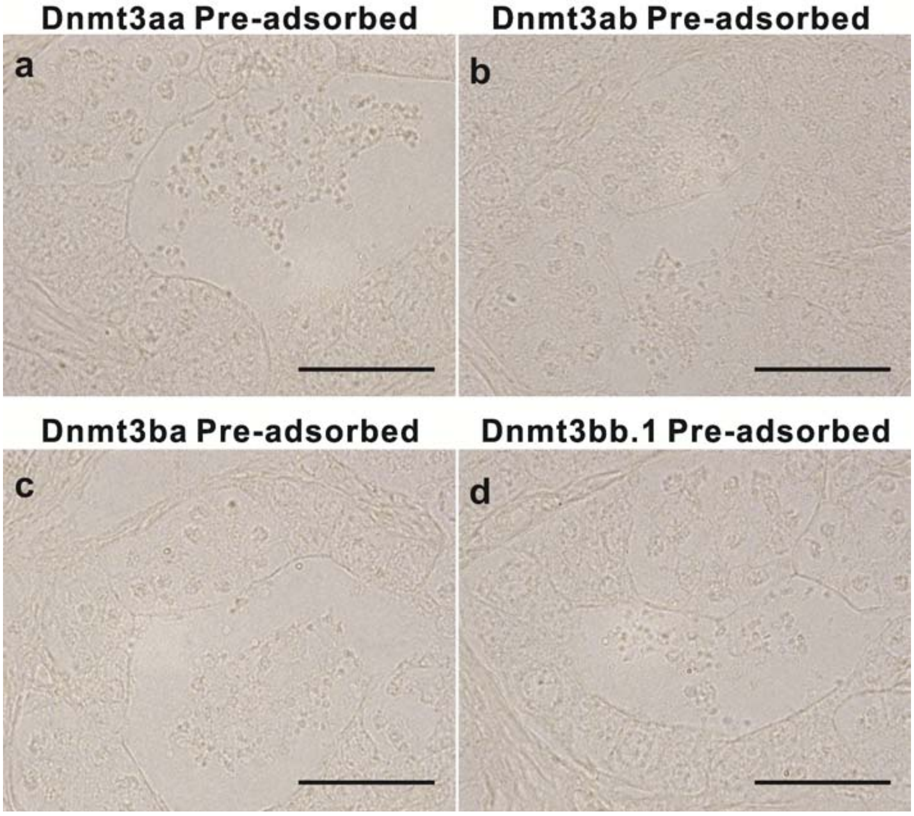

Supplementary Figure S5. Negative controls for Dnmt3aa (a), Dnmt3ab (b), Dnmt3ba (c), and Dnmt3bb.1 (d) immunoreactivities in mature testes of ricefield eels. The sections from the same testicular tissues as shown in FIG. 3 were immunoreacted with anti-Dnmt3aa, anti-Dnmt3ab, anti-Dnmt3ba, and anti-Dnmt3bb.1 antisera pre-adsorbed by excessive Dnmt3aa-N, Dnmt3ab-N, Dnmt3ba-N, and Dnmt3bb.1-N, respectively, and visualized by DAB chromogen. Scale bar = 25 μm.

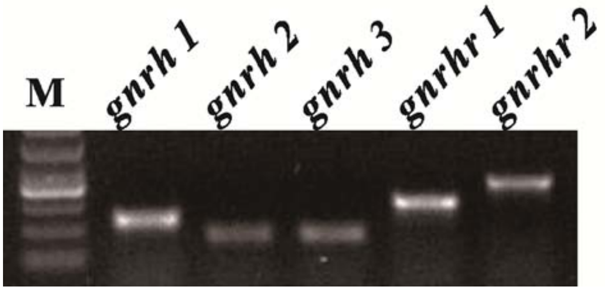

Supplementary Figure S6. RT-PCR analysis of *gnrh* and *gnrhr* mRNA expression in testes of ricefield eels. The genes analyzed were shown above the corresponding lanes. M, DL1000 DNA Ladder Marker (Takara, Dalian, China). Total RNA was isolated from testes of ricefield eels using TRIzol (Invitrogen), and treated with DNase I (1U/μl) to remove any genomic DNA contamination. Then 1 μg of total RNA was reverse transcribed with random primers using the RevertAid^{TM} H Minus First Strand cDNA Synthesis Kit (Thermo Scientific) according to the manufacture's instruction. The first-strand reaction was amplified for each target gene using the TGRADIENT thermocycler (Biometra GmbH, Goettingen, Germany). PCR was performed in a 25-μl final volume containing 2.5 μl 10xTaq buffer, 2.0 mM $MgCl_2$, 0.2 mM dNTP, 0.4 μM of each primer, and 1.25 U Taq DNA Polymerase (Thermo Scientific). The primer sets were GnRH1-F/GnRH 1-R, GnRH2-F/GnRH 2-R, GnRH3-F/GnRH 3-R, GnRHR-I-F/GnRHR-I-R, and GnRHR-II-F/GnRHR-II-R for ricefield eel *gnrh 1* (AY858056), *gnrh 2* (AY858054), *gnrh 3* (AY858055), *gnrhr 1* (KX524496) and *gnrhr 2* (KX524497), which generated PCR fragments of 247, 182, 174, 314, and 406 bp, respectively. The sequences of PCR primers are as follows: GnRH1-F, 5'-ACTGTGGCTGCTGCTTGTGG -3'; GnRH1-R, 5'-GTGTCCGTTTTCCGTGTCAGTG-3'; GnRH2-F, 5'-TGATTCGGCTGGTCT TGCTG-3'; GnRH2R, 5'-TCTGAGTATGCTCCTCCTCTGGG-3'; GnRH3-F, 5'-GTTGTTG GCGTTGGTGGTTC-3'; GnRH3-R, 5'-GGTCTAAGTCTCTGTTGGATTGGG-3'; GnRHR-I-F, 5'-CAACCCTCTGGCTATCAGTAAAG-3'; GnRHR-I-R, 5'-GCACTTCATTTG AGGGCCAGTTA-3'; GnRHR-II-F, 5'-TGCTCTGTAGGCTGCTCTGCTTC-3'; GnRHR-II-R, 5'-ATGACTCACCTGCTTTATCCCTC-3'. The PCR products were separated on a 2% agarose gel and visualized by staining with ethidium bromide.

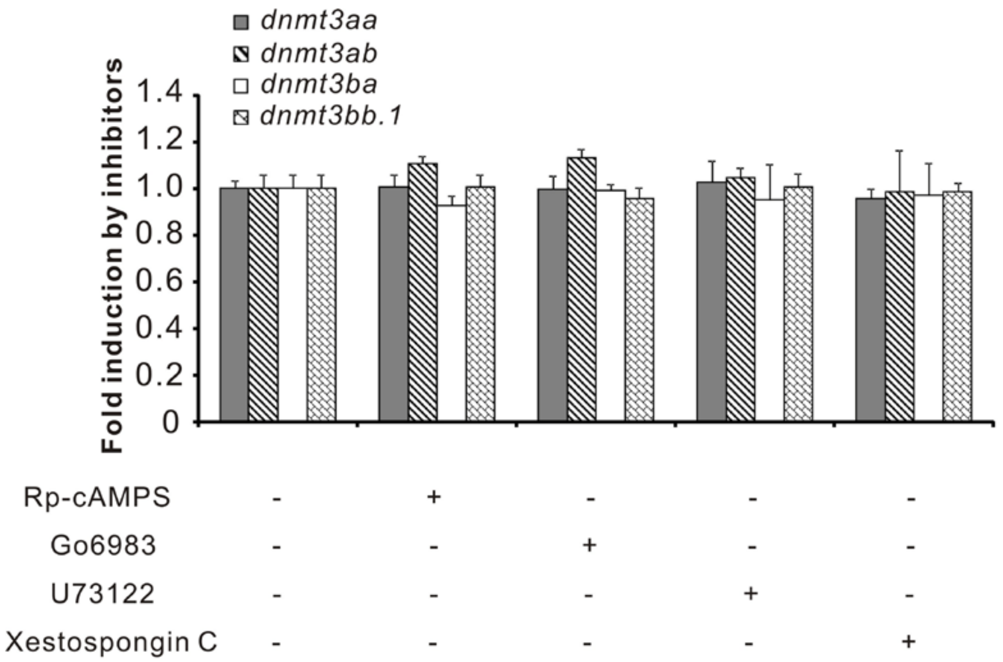

Supplementary Figure S7. Effects of inhibitors of intracellular signaling pathways on the expression of *dnmt3aa*, *dnmt3ab*, *dnmt3ba*, and *dnmt3bb.1* in the *in vitro* incubated testicular fragments of ricefield eels. The testicular fragments were pre-incubated for 18 h before treating with the inhibitor Rp-cAMPS (50 μM), Go6983 (10 μM), U73122 (10 μM) or Xestospongin C (1 μM) respectively for 8 h. After treatment, mRNA levels of *dnmt3aa*, *dnmt3ab*, *dnmt3ba*, and *dnmt3bb.1* in the testicular fragments were quantified with real-time quantitative PCR and were presented as fold change relative to the vehicle control. Each bar represents mean ± SEM of 6 replicates. No significant differences were observed between treatments (*P*>0.05).

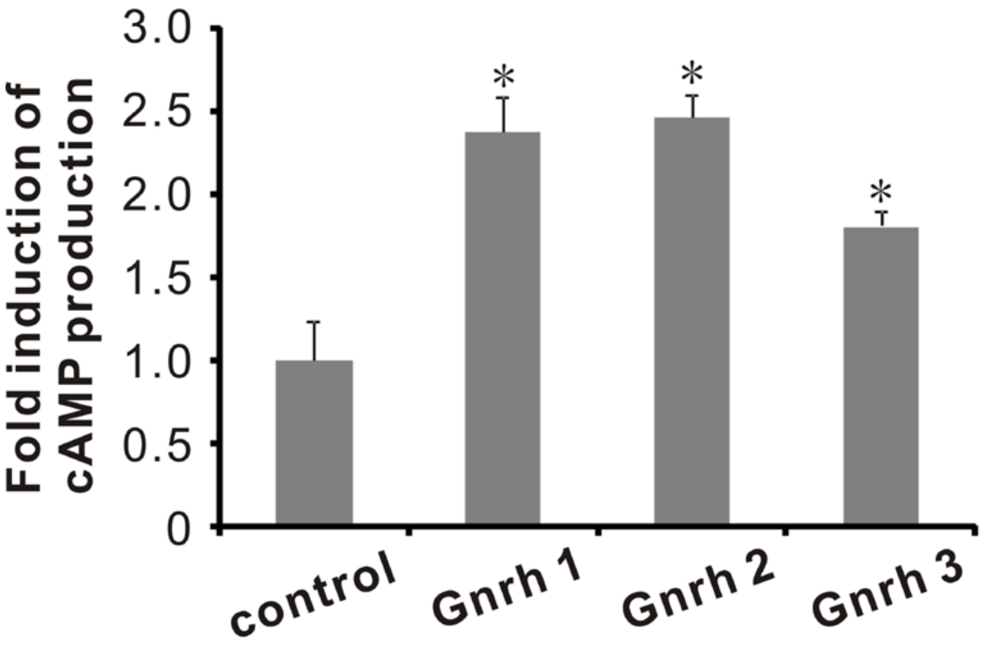

Supplementary Figure S8. The effects of Gnrh on cAMP production in the in vitro incubated testicular fragments of ricefield eels. The testicular fragments were pre-incubated for 18 h before treating with Gnrh 1, Gnrh 2, or Gnrh 3 (100 nM) for 8 h. After treatment, the amount of cAMP in the testicular fragments was quantified with a Monoclonal Anti-cAMP Antibody Based Direct cAMP ELISA Kit (80203, NewEast Biosciences, Inc., PA, USA). Results are expressed as fold induction relative to the vehicle control. Each bar represents the mean ± SEM of 6 replicates. *$P<0.05$ *vs* the vehicle control.

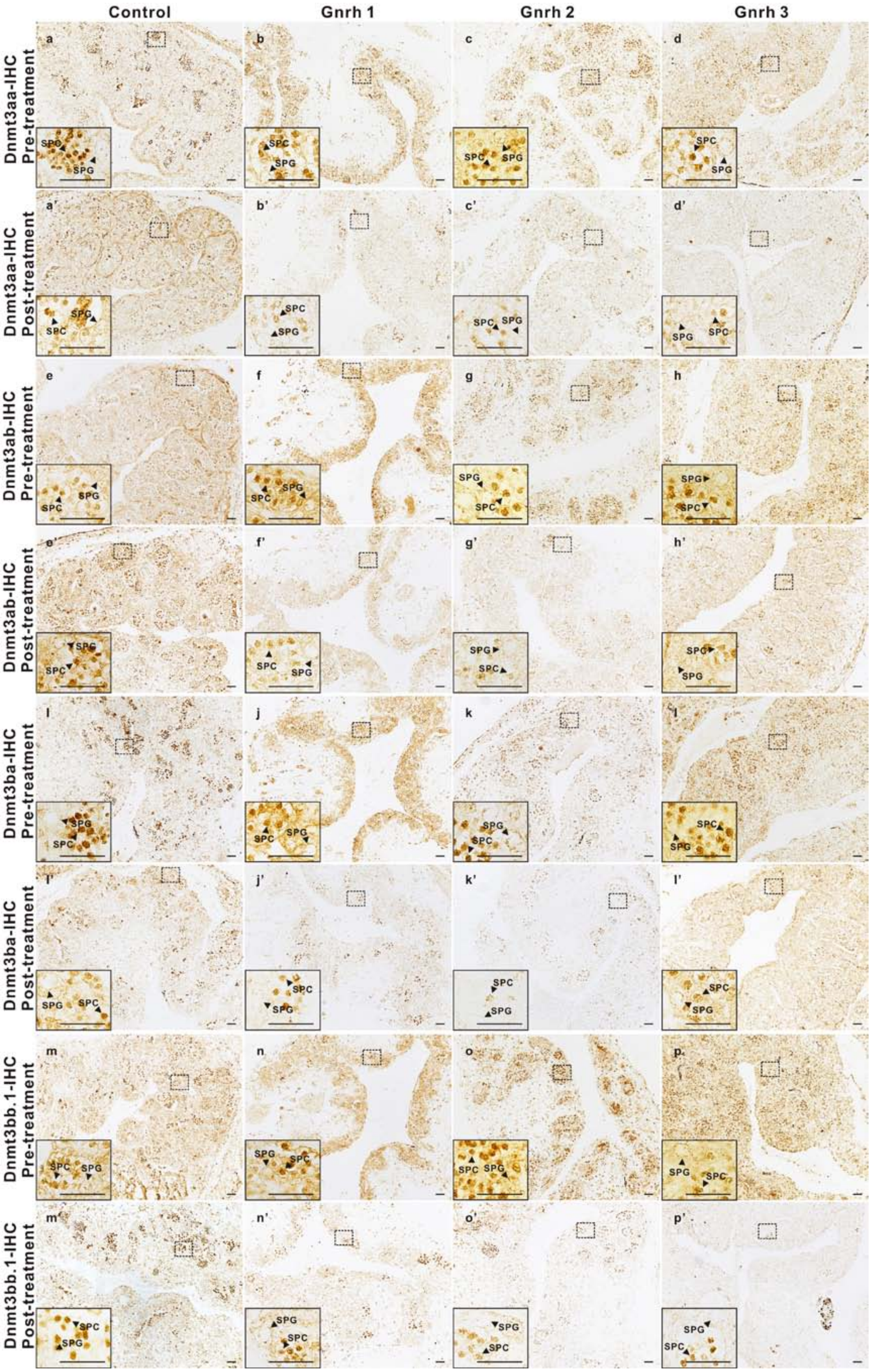

Supplementary Figure S9. Immunostaining for Dnmt3aa (a~d; a'~d'), Dnmt3ab (e~h; e'~h'),

Dnmt3ba (i~l; i'~l'), and Dnmt3bb.1 (m~p; m'~p') in testes of ricefield eels before (pre-treatment, a~p) and after (post-treatment, a'~p') intraperitoneal injections of Gnrh 1, Gnrh 2, and Gnrh 3. The male ricefield eels were first examined by biopsy and then treated with Gnrh 1, Gnrh 2 and Gnrh 3 (0.1 μg/g body weight) twice a week for four weeks. After treatment, the expression of Dnmt3 proteins in testes was detected by immunohistochemistry. The anti-Dnmt3aa (1:200), Dnmt3ab (1:200), Dnmt3ba (1:200) or Dnmt3bb.1 (1:200) antiserum was used as the primary antiserum. The secondary antibody was 1:500 diluted horseradish peroxidase (HRP)-conjugated goat anti-mouse IgG H+L (115-035-003, Jackson ImmunoResearch Laboratories, Inc.). The immunoreactive signals were visualized by DAB chromogen. The insets are higher magnification of the boxed areas within each image. SPC: spermatocyte; SPG: spermatogonium. Scale bar = 25 μm.

Supplementary Table S1. Sequences of oligonucleotide primers used in cloning of *dnmt3*

Primer Name	Sequence (5' to 3')
eDnmt3-F_1	AYGGYTAYCAGTCBTACTGCAC
eDnmt3-F_2	CTDTTTGCTGGHATHGCNACAGG
eDnmt3-R_1	CKGATGACNGGNACRCTCCARGA
eDnmt3-R_2	CCNGGHAKGTTNCCCCAGAAGTA
eDnmt3aa-F_1	CGACGGCTACCAATCCTACTG
eDnmt3aa-F_2	TCCCTGTCTACATGGACAACAA
eDnmt3aa-F_3	ACBTACAACAARCAGCCCRTKTAC
eDnmt3aa-F_4	TGAAWGAAGCTGCACCTGGTGC
eDnmt3aa-R_1	CCGCTCGTCATCTGGCTTTGG
eDnmt3aa-R_2	TTCTGCCACAACTGGAGGATA
eDnmt3aa-R_3	ACTGGCTGGCTCCTCCTCTTT
eDnmt3aa-R_4	TTTCCGACGCCCACGCTTCTT
eDnmt3ab-F_1	CATGGGTGTCAGTGATAAGAGG
eDnmt3ab-F_2	AGGACTGTTTAGAGCATGGCAG
eDnmt3ab-F_3	GAGGGMKVYCGDGGSMGGCTGCG
eDnmt3ab-F_4	CATCYSTCRTTKCYWTMTTTTC
eDnmt3ab-R_1	ATCAAATAGCGACAGGACACGA
eDnmt3ab-R_2	AGTGCGACGCTCCAACAGACCA
eDnmt3ab-R_3	CTGCTGCTTTGAAGGCTGTGGG
eDnmt3ab-R_4	CTCCATCTTCCACTTGGTCAGC
eDnmt3ba-F_1	TTACCGGATACTGACCTTGTTG
eDnmt3ba-F_2	CCAGGATTGTTTGGAGGTTGGA
eDnmt3ba-F_3	TKYTTTYCTASMGARSTGCAAG
eDnmt3ba-F_4	TGTGKTVCTDKYKGGVTTTTTTGA
eDnmt3ba-F_5	RRATGARTTYYTKGAGTAYCAG
eDnmt3ba-R_1	GGCAGGGATAGAGGGATAAACT
eDnmt3ba-R_2	TCCAGGGATCAACATCTTTCAG
eDnmt3ba-R_3	TTCTCTCCAGGTACGTGGTCAT
eDnmt3ba-R_4	CATTAAGGTTATTCGGTTCGCT
eDnmt3ba-R_5	CAAGCAGCATCAGCAATAGTGTA
eDnmt3ba-R_6	GCTAAGTTCCCTGAGCTGTGAG
eDnmt3bb.1-F	TTGGATGAGTGCAAGAAGCGTT
AP	GGCCACGCGTCGACTAGTAC[T]16
BRL-A_2	GGCCACGCGTCGACTAGTAC

F: sense primer; R: antisense primer.

Supplementary Table S2. Sequences of oligonucleotide primers used in quantitative PCR analysis of *dnmt3* and production of recombinant Dnmt3 polypeptides.

Primer Name	Sequence (5' to 3')
qrt-ednmt3aa-F	CGGAAACTAGAGCAAACCGA
qrt-ednmt3aa-R	TGGAAAGTCACCCTTGGGAC
qrt-ednmt3ab-F	GAAACTGGAGCGCCCTACGA
qrt-ednmt3ab-R	CTCTCTGGTCCGCTTGCTAA
qrt-eDnmt3ba-F	AGCAAGTATGACGTCATGTC
qrt-eDnmt3ba-R	AGGTACGTGGTCATTTTCAT
qrt-ednmt3bb.1-F	CGAATGGTGAATGAAGTTCTGA
qrt-ednmt3bb.1-R	CAAGTACACATCCTTGCAGGTT
b-actin-qF_2	GCAGAGCCTAGACGACCAACTC
b-actin-qR_2	GGGTGCGTTTCTTAAACCTAGC
gapdh-qF	TCACTGCTACCCAGAAGACCG
gapdh-qR	CTCAGGAATGACCTTGCCCAC
hprt1-qF	TTGGACAGGACAGAGCGACT
hprt1-qR	TCATTGGGATGGAGCGGT
eDnmt3aa-pET-F	GAGACCATGGGCATGCCGTCAAACTGTCCTGC
eDnmt3aa-pET-R	GAGAGGATCCCTACTGGTACTCAATCTCACTGG
Exp-ednmt3aa-F	GAGAGGATCCATGCCGTCAAACTGTCCTGC
Exp-ednmt3aa-R	GAGACTCGAGCTACAGTAAGCCGTATGTGCTCC
eDnmt3ab-pET-F	GAGACCATGGGCACAATCCCGGACATGGACCC
eDnmt3ab-pET-R	GAGAGGATCCCTAGAAGGTTATTCTGGGCATCG
Exp-ednmt3ab-F	GAGAGGATCCATGCCGTCCAACACTGTCAC
Exp-ednmt3ab-R	GAGACTCGAGCTATGTGTAGGCTGCTGCTTCTG
eDnmt3ba-pET-F	GAGACCATGGGCTACGACCCTGTGAAAGCTCG
eDnmt3ba-pET-R	GAGAGGATCCCTATGCAATATCTTTGGGGTAGC
Exp-ednmt3ba-F	GAGAGGATCCATGGCGAGTACAGCGGTTGT
Exp-ednmt3ba-R	GAGACTCGAGCTACTTGGAGAAGATTAACTTGG
eDnmt3bb.1-pET-F	GAGACCATGGGCATGGTTATGTTTGAAAAGGA
eDnmt3bb.1-pET-R	GAGAGGATCCCTATGGCTGGATTTCAGAGCTGG
Exp-ednmt3bb.1-F	GAGAGGATCCATGGTTATGTTTGAAAAGGA
Exp-ednmt3bb.1-R	GAGACTCGAGCTACAGACAGTCCACGCAGAAAC

F: sense primer; R: antisense primer
